# Disorder-Specific Genetic Effects Drive the Associations Between Psychopathology and Cognitive Functioning

**DOI:** 10.1016/j.bpsgos.2025.100680

**Published:** 2025-12-18

**Authors:** Wangjingyi Liao, Engin Keser, Andrea Allegrini, Kaili Rimfeld, Robert Plomin, Margherita Malanchini

**Affiliations:** a Centre for Brain and Behaviour, School of Biological and Behavioural Sciences, Queen Mary University of London, London, United Kingdom; b Social, Genetic and Developmental Psychiatry Centre, King’s College London, London, United Kingdom; c Division of Psychology and Language Sciences, University College London, London, United Kingdom; d Department of Psychology, Royal Holloway University of London, London, United Kingdom

**Keywords:** Cognitive development, Cognitive functioning, Polygenic scores, Psychopathology, Transdiagnostic genetic risk, Within-siblings analyses

## Abstract

**Background:**

Cognitive functioning is a critical dimension of psychopathology that remains underinvestigated. Because cognitive deficits often transcend diagnostic boundaries, it has been challenging to delineate specific relationships between psychiatric disorders and cognitive functioning. Genetic research offers novel, powerful tools to disentangle shared (transdiagnostic) from disorder-specific effects, thereby opening new avenues for understanding how psychopathology relates to cognition.

**Methods:**

We used genomic structural equation modeling to identify transdiagnostic and disorder-specific genetic risk. We derived polygenic scores in a longitudinal twin sample (*N* = 7764) to examine associations between genetic risk for psychopathology and domains of cognitive functioning across development.

**Results:**

The results showed that relationships between psychopathology and cognition are primarily driven by disorder-specific genetic risk rather than by transdiagnostic factors. Associations differed across disorders and cognitive domains. Within-family analyses indicated that associations between genetic risk for psychopathology and cognition operated through distinct pathways. While for some disorders (e.g., attention-deficit/hyperactivity disorder), the effects could be attributed to confounding by environmental differences between nuclear families, for others (e.g., autism spectrum disorder), effects were robust to environmental confounding.

**Conclusions:**

In contrast to psychiatric symptoms, which are most effectively predicted by transdiagnostic genetic risk, our findings highlight the need to consider disorder-specific genetic effects when examining associations between psychopathology and cognition. Focusing solely on transdiagnostic risk is unlikely to capture the full complexity of these relationships or enhance our understanding of the distinct cognitive profiles associated with psychopathology.

## Plain Language Summary

Cognitive functioning is integral to psychiatric conditions, but the genetic underpinnings of this relationship remain unclear. We examined how genetic liability to multiple psychiatric disorders relates to cognitive outcomes across development. Leveraging polygenic score analyses and longitudinal twin data, we disentangled shared genetic effects from disorder-specific influences. Our analyses revealed that most associations between psychiatric risk and cognitive ability are driven by disorder-specific genetic factors rather than a common transdiagnostic architecture. These effects varied across disorders and appeared to operate through distinct developmental pathways. These findings underscore the need to consider disorder-specific genetic architectures when investigating cognitive differences in psychiatry.

Cognitive development is characterized by age-related increases in several cognitive functions, including vocabulary, complex reasoning, and learning. Variation in cognitive development is associated with key societal and medical outcomes throughout the lifespan, including physical and mental health (1,2). Studies have highlighted the positive association between cognitive functioning and mental health outcomes across the lifespan (3): Higher cognitive competencies have been consistently associated with better mental health (4). An association between cognition and mental health is also observed at the other end of the spectrum of health and ability, as cognitive deficits are common in patients diagnosed with psychiatric disorders (5).

Despite this well-established association between cognition and mental health, cognitive functioning remains an underinvestigated dimension of psychopathology compared with affective and emotional symptoms (6). Cognitive deficits often transcend traditional diagnostic boundaries in the same way that core affective and emotional symptoms are shared across multiple conditions (7). For example, an inability to forget or inhibit thoughts is central to both posttraumatic stress disorder (PTSD) and obsessive-compulsive disorder (OCD), where patients experience intrusive thoughts or intense flashbacks. Cognitive deficits in attention, memory, and language are also observed across schizophrenia (SCZ), bipolar disorder (BIP), and autism spectrum disorder (ASD) [see Millan *et al.* (6) for a review].

This overlap between cognitive symptoms across psychiatric disorders makes investigating specific relationships between cognition and mental health, as well as their origins, difficult. It is likely that specific aspects of psychopathology will impact cognitive skills differently, and specific cognitive profiles will lead to different psychopathological symptoms. A further challenge associated with investigating developmental effects is the heterogeneity in the average age of onset and diagnosis of different psychiatric conditions, which ranges widely from childhood (e.g., ASD and attention-deficit/hyperactivity disorder [ADHD]) to adolescence (e.g., anorexia nervosa [AN], anxiety [ANX], and depression) and early adulthood (e.g., SCZ and BIP).

One way of overcoming these challenges is to consider genetic risk. Genetic studies provide a powerful tool for uncovering and isolating shared and unique processes to each disorder. Research has shown that both cognitive abilities and psychiatric conditions are significantly heritable (8,9), although heritability estimates vary widely across disorders, with some disorders, such as SCZ and ADHD, evincing higher heritability estimates than others, such as depression and anxiety (10, 11, 12). Studies have suggested that shared genetic factors may account for part of the associations between cognitive abilities and psychopathology. For example, a twin study found that up to 40% of the observed association between symptoms of psychopathology and cognitive ability measured in early childhood could be attributed to shared genetic influences (13). Analyses of genome-wide association data also showed that genetic risk for psychopathology is significantly associated with cognition-related traits such as educational attainment (12).

Studies investigating the association between psychopathology and cognitive functioning have largely focused on the general psychopathology factor, also called the p-factor (14). The p-factor is thought to capture transdiagnostic effects across psychiatric conditions, and as such, it offers a useful framework for investigating generality and specificity in the development of the association between cognition and psychopathology. Recent findings suggest that the p-factor also operates at a genetic level (12,15,16) and that removing transdiagnostic genetic effects can reveal nuanced associations with other traits, including cognition (13,17,18). For example, isolating transdiagnostic effects significantly changed the genetic correlations between psychiatric disorders and educational attainment, which increased significantly for SCZ and BIP and decreased significantly for depression (17). These findings highlight the importance of separating transdiagnostic from disorder-specific genetic factors to better understand the association between psychopathology and cognitive development.

Thus, in this study, we aimed to investigate the complex relationship between psychopathology and cognitive development by leveraging genetic data to isolate transdiagnostic risk factors and explore the generality and specificity of these associations. Using polygenic scores (PGSs), which combine trait-associated genetic variants into a single composite index (19), in a large longitudinal sample, we investigated the association between transdiagnostic and disorder-specific genetic risk for psychopathology and cognitive development from ages 4 to 23 years. We examined how genetic risk for psychopathology is associated with differences in general cognitive ability, as well as with the development of verbal and nonverbal reasoning from childhood to early adulthood.

Finally, using family-level data, we explored the potentially different genetic pathways underlying the association between psychopathology and cognition. Investigating PGS associations within a sibling-difference design allowed us to look at associations free from several potential confounding factors, including genetic (e.g., assortative mating, population stratification) and environmental influences shared by family members (e.g., passive gene-environment correlation) and therefore capture more direct genetic effects.

Studies that have examined the association between genetic risk for psychopathology and psychiatric symptoms have found that these effects largely operate through direct pathways. For example, studies have found a significant direct genetic association between genetic risk for externalizing behavior and conduct problems/ADHD-related symptoms (20). Genetic risk for neurodevelopmental conditions was also found to be associated with language, motor, social, and communication skills largely via direct effects (21). These studies have primarily focused on investigating within-trait prediction, examining the associations between genetic risk for psychiatric disorders and manifestations of psychopathology. However, cross-trait associations—specifically, the pathways linking genetic risk for psychiatric disorders to cognitive profiles over development—remain unexplored. By integrating developmental and genetic frameworks, in this study, we seek to advance our understanding of the association between psychopathology and cognitive functioning.

## Methods and Materials

### Preregistration

The methods, hypotheses, and analyses were preregistered on Open Science Framework (https://osf.io/7enr5/).

### Sample

Participants were drawn from the TEDS (Twins Early Development Study) sample, a longitudinal study that recruited more than 15,000 twin pairs born in England and Wales between 1994 and 1996. Currently, approximately 10,000 families remain actively involved in TEDS, nearly 30 years after the first wave of data collection. TEDS participants and their families remain largely representative of the UK population for their birth cohort (22,23). In terms of ethnicity and socioeconomic status (SES), the majority of the sample (93%) identified as White British; 48.7% of the sample held a university degree, a higher percentage than the national estimate of 29% for that birth cohort; and 52.7% of participants were employed at age 21, comparable to the national estimate of 63.4% (22). More details about the representativeness of the TEDS sample are available elsewhere (22).

This study used TEDS data collected across 7 waves, when the twins were ages 4, 7, 9, 12, 16, 19, and 23 years. Genotyping was conducted in 2 separate waves, 5 years apart, using 2 different platforms: Affymetrix GeneChip 6.0 single nucleotide polymorphism (SNP) arrays and Illumina HumanOmniExpressExome-8v1.2 arrays. After quality control, genotypes from 10,346 samples were retained, comprising 3320 dizygotic (DZ) twin pairs and 7026 unrelated individuals. The final dataset contained 7,363,646 genotyped or well-imputed SNPs.

Individuals with both genetic data and cognitive abilities measured at at least 1 time point were included in the current analyses, resulting in final analytic samples ranging from 1180 to 7764 individuals, depending on the collection wave and cognitive abilities (Table S1). Given the low cross-ancestry portability of PGSs, our analyses were restricted to participants of European ancestry. Further details on DNA collection, genotyping procedures, and quality control are provided elsewhere (16,24).

Written parental consent was obtained from all participants before data collection at every wave and from the twins at ages 16, 19, and 23 years. We excluded participants with severe medical conditions or uncertain/unknown zygosity from the analyses. TEDS received ethical approval from the research ethics committee of King’s College London (reference IDs: PNM/09/10–104 and HR/DP-20/21–22060).

### Measures

Here, we provide a brief description of all the variables included in the current study. For detailed descriptions of each subscale and items, refer to https://www.teds.ac.uk/datadictionary.

### Measuring Genetic Risk for Psychopathology

We calculated genetic risks for psychopathology using summary statistics from 11 genome-wide association studies (GWASs) of major psychiatric disorders (12): ADHD (25), ASD (26), PTSD (27), OCD (28), ANX (29), major depressive disorder (MDD) (30), SCZ (31), BIP (32), AN (33), alcohol dependence (ALCH) (34), and Tourette syndrome (TS) (35). The current sample was not included in any of these GWASs.

The transdiagnostic psychopathology factor (the p-factor) GWASs and the residual GWASs, obtained after isolating the genetic variance captured by the p-factor, were derived using summary statistics from a previous study that used genomic structural equation modeling to investigate generality and specificity in psychiatric disorders using the same 11 GWASs (17).

A common factor model was used to estimate SNP effects that are shared across all 11 psychiatric disorders (the p-factor). Meanwhile, the effect estimates of each SNP on the residual variance from each psychiatric disorder GWAS that was not explained by the common factor was obtained. These residual effects for each SNP indicate specific genetic effects on each psychiatric disorder beyond what is shared among them. Therefore, these residual effects were referred to as non-p genetic effects. The summary statistics for the p-factor as well as the 11 non-p psychiatric disorders were then used to calculate PGSs in the independent TEDS cohort. Details of the summary statistics, including cohorts, sample sizes, and heritability estimates based on linkage disequilibrium score regression are available in Tables S10 and S11.

### PGSs in TEDS

PGSs were created using a standard pipeline applied to all PGSs created in TEDS. The PGS pipeline is based on the LDPred2 auto option using the R package bigsnpr (36). Default parameters were used in the standard pipeline as recommended in the LDpred2 manual. Analyses were restricted to HapMap3 variants and based on precomputed LD matrices from the UK Biobank. Compared with other methods and previous iterations (i.e., LDpred1), LDpred2 offers higher predictive power and overcomes previous limitations, including a more efficient handling of long-range LD regions (36).

Standard genetic confounding variables, including the top 10 principal components of genetic ancestry, genotyping batch and chip, sex, and age, were included in all analyses.

### Measures of Cognitive Abilities

Cognitive abilities were measured using standardized cognitive tests collected at ages 4, 7, 9, 12, 16, 19, and 23 years. These composite scores were designed to accommodate children’s developmental stages and were derived from different questionnaires at each age. At each time point, 3 composite scores were calculated: verbal cognitive ability, nonverbal cognitive ability, and general cognitive ability (g). Composite scores for verbal and nonverbal reasoning were derived from the mean of the standardized tests described below. The standardized g composite was derived by taking the mean of the standardized verbal and nonverbal composite scores.

#### Age 4

Cognitive abilities were assessed using 4 tests administered as booklets sent to parents to complete. Two tests assessed verbal reasoning: the 48-item MacArthur Communicative Development Inventories (37) and an ordinal grammar composite scale. Nonverbal reasoning was assessed using the parent report and parent-administered Parental Report of Children’s Ability questionnaire, which was developed by the TEDS team based on a battery of scales (38).

#### Age 7

Cognitive ability was measured using 4 child-completed tests administered over the telephone by trained research assistants, including 2 verbal tests (a 13-item similarity test and an 18-item vocabulary test), both derived from the Wechsler Intelligence Scale for Children, Third Edition (WISC-3) (39), and 2 nonverbal tests [a 9-item conceptual groupings test from the McCarthy scales (40) and a 21-item WISC picture completion test (39)].

#### Age 9

Cognitive ability was assessed using 4 tests that were administered as booklets sent to families to be completed by the children. Verbal reasoning was assessed using 2 tests, the first 20 items of a word test and the first 18 items of a general knowledge test from WISC-3-PI (41). Nonverbal reasoning was assessed using the shapes and puzzle tests from Cognitive Abilities Test 3rd edition (42) figure classification and figure analogies.

#### Age 12

Cognitive ability was assessed using 4 tests that were administered online and completed by the children. Verbal reasoning was assessed using the 30-item full version of the word test and the general knowledge test from WISC-3-PI (41). Nonverbal reasoning was assessed using the pattern test adopted from Raven’s standard progressive matrices (43) and the picture completion test adopted from WISC-3-UK (44).

#### Age 16

Cognitive ability was assessed using 2 tests administered online and completed by the children. Verbal reasoning was assessed using the Mill Hill vocabulary test (44), and nonverbal reasoning was assessed using Raven’s standard progressive matrices test (43).

#### Age 19

Spatial abilities were measured separately. They were administered when the sample was aged 19 to 22 through 2 online batteries: King’s Challenge and Spatial Spy. The first battery measures spatial manipulation, while the second focuses on spatial navigation. Details of battery development and its tests have been outlined elsewhere (45,46).

#### Age 23

Cognitive skills were measured using Pathfinder (47), a brief, gamified measure of general, verbal, and nonverbal cognitive abilities developed and validated by the TEDS team.

### Additional Measures

Four cognition-related PGSs and family SES were included in robustness check analyses (see Statistical Analysis). The 4 cognitive PGSs were cognitive and noncognitive abilities (48), a genomic general cognitive ability factor [genomic g (49)], and intelligence (50). The 4 cognitive PGSs were selected based on their power and representativeness.

Family SES was measured using a composite of 5 variables: mother and father employment levels, mother and father educational levels, and mother’s age at the birth of her first child. The SES composite score was the most often used SES variable in the TEDS and showed great stability across development (23).

### Statistical Analysis

#### Regression of Cognitive Abilities on PGSs

Multiple regression was used to test for predictions of psychopathology PGSs on cognitive ability scores. We used the total sample in our analysis and accounted for nonindependence of observations for DZ co-twins using the generalized estimating equation. False discovery rate (FDR) correction (51) was applied to adjust the criteria for significance. Differences in PGS predictions before and after correcting for *p* and differences between verbal and nonverbal cognitive abilities were tested for significance using the *z* score test.

#### Within- and Between-Family Analyses

We used a sibling-difference design in DZ twins to account for family-fixed effects by examining differences between siblings, thereby isolating differences between siblings in genetic variation from between-family factors (52).

Because sibling genotypes differ randomly due to meiosis, each sibling has an equal probability of inheriting any given parental allele. Consequently, within-sibling PGS associations are less confounded by between-family environmental variation, including population stratification; indirect genetic effects—associations between nontransmitted parental alleles and offspring outcomes operating via the rearing environment; or demographic factors such as assortative mating (53). Therefore, differences in PGS associations observed within sibling pairs are interpreted as reflecting more direct genetic effects, whereas between-family associations likely capture a combination of indirect genetic influences and shared environmental effects.

#### Robustness Check

We reran the multiple regression analyses with additional covariates to test the robustness of the results, including family SES and cognitive PGSs (see Measures). Each robustness check variable was included in the multiple regression analysis separately.

#### Cognitive Change Analysis

In Supplemental Note, we present the method and results of our preregistered analysis examining cognitive change scores of general, verbal, and nonverbal abilities.

## Results

### Psychopathology and General Cognitive Abilities

**Figure 1:**
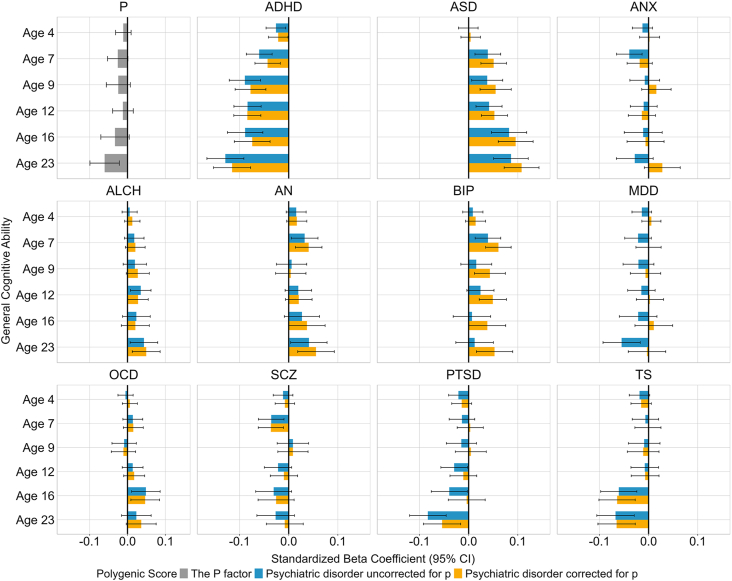
Associations between polygenic scores for psychiatric disorders and general cognitive ability across development. The gray bars represent the associations (standardized beta coefficient) between transdiagnostic genetic risk for psychopathology (p-factor) and general cognitive ability over development from ages 4 to 23 years. The blue and yellow bars show associations between genetic risk for each psychiatric disorder and general cognitive ability over development before (blue) and after (yellow) removing the effects of transdiagnostic genetic risk. The error bars indicate 95% CIs. ADHD, attention*-*deficit*/*hyperactivity disorder; ALCH, alcohol dependence; AN, anorexia nervosa; ANX, anxiety; ASD, autism spectrum disorder; BIP, bipolar disorder; MDD, major depressive disorder; OCD, obsessive-compulsive disorder; PTSD, posttraumatic stress disorder; SCZ, schizophrenia; TS, Tourette syndrome.

Figure 1 presents the results of the multiple regression analyses examining the association between genetic risk for psychopathology, measured using PGSs, and general cognitive ability from ages 4 to 23 years. Transdiagnostic genetic risk, reflected in the p-factor PGS, was minimally associated with general cognitive ability across development. The only significant association was observed at age 23 (β = −0.060, SE = 0.020, *p*_FDR_ = .012). Consequently, the effect sizes of the predictions remained virtually unchanged when comparing disorder-specific PGSs before and after accounting for transdiagnostic effects (see Figure 1 blue vs. yellow bars).

Associations between genetic risk for psychopathology and general cognitive ability were negative for some disorders (e.g., ADHD, TS, and PTSD) and positive for others (e.g., ASD, AN, and BIP); however, some did not survive FDR correction (see Table S2). Robustness checks showed that most associations between psychiatric PGSs and general cognitive ability remained significant after controlling for family SES or cognition-related PGSs by means of multiple regression (Table S2).

Interestingly, associations between individual psychiatric disorders and general cognitive ability were developmentally consistent for each disorder, and for some disorders (e.g., ADHD and ASD), effect sizes increased across development. Particularly, the ADHD PGS showed the strongest association across development, ranging from β = −0.026, SE = 0.010, *p*_FDR_ = .038 at age 4 to β = −0.129, SE = 0.0192, *p*_FDR_ < .001 at age 23. The ASD PGS also showed significant associations with general cognitive ability across development (ranging between β = 0.039, SE = 0.013, *p*_FDR_ = .015 at age 7 and β = 0.086, SE = 0.018, *p*_FDR_ < .001 at age 23), except for during early childhood (β = −0.0007, SE = 0.010, *p*_FDR_ = .95).

Given the observed developmental increase in the association between PGSs and general cognitive ability, we examined whether this pattern of results could be due to measurement instability across development or an actual increase in genetic effects. We compared 2 alternative models. First, we tested the PGS prediction of a common factor model constructed including all measures of general cognitive ability across development as indicators. Second, we tested a specific effects model in which the PGS predicted the residual variance specific to each cognitive assessment that was not captured by the common g factor (54).

We tested these 2 alternatives including the ADHD and the ASD PGSs and found that, for both PGSs, the specific effects model provided a significantly better fit than the common factor model, suggesting that the developmental pattern was better explained by specific genetic associations between psychopathology-related PGSs and cognitive outcomes than by measurement instability (ADHD and g: χ^2^_5_ = 17.37, *p* = .004; ASD and g: χ^2^_5_ = 18.54, *p* = .002). To test whether the PGS prediction was significantly different across developmental stages, we constrained PGS effects to be equal across waves and compared the fit of this model with a model that allowed PGS effects to vary freely across waves. We found that this second model was a significantly better fit for both ADHD (χ^2^_5_ = 43.645, *p* < .001) and ASD (χ^2^_5_ = 30.14, *p* < .001), indicating that PGS effects on cognition become significantly stronger over developmental time (Tables S12 and S13). All models were estimated using full information maximum likelihood to account for sample attrition across assessment waves.

### Psychopathology and Specific Cognitive Abilities

**Figure 2:**
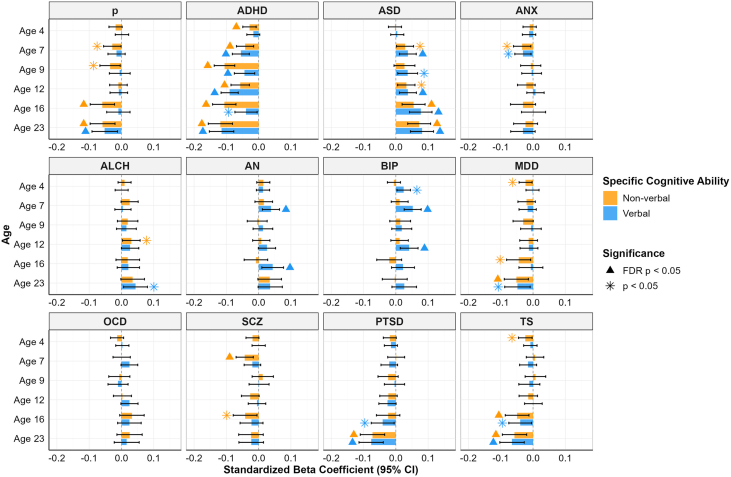
Associations between polygenic scores for psychiatric disorders and verbal and nonverbal reasoning across development. The x-axis shows the standardized beta coefficient, and the y-axis shows the cognitive outcome at ages 4 to 23 years. Each panel shows results for a different psychiatric disorder. The yellow bars are associations with nonverbal reasoning, and the blue bars are associations with verbal reasoning. Error bars indicate 95% CIs. The stars indicate significance at a threshold of *p* < .05, and the triangles indicate significance after FDR correction. ADHD, attention-deficit/hyperactivity disorder; ALCH, alcohol dependence; AN, anorexia nervosa; ANX, anxiety; ASD, autism spectrum disorder; BIP, bipolar disorder; FDR, false discovery rate; MDD, major depressive disorder; OCD, obsessive-compulsive disorder; PTSD, posttraumatic stress disorder; SCZ, schizophrenia; TS, Tourette syndrome.

To further explore the association between psychopathology and cognition, we extended our analyses to examine the prediction from psychiatric PGSs to 3 more specific subcomponents of cognitive functioning: verbal reasoning, nonverbal reasoning, and spatial ability. Figure 2 presents the psychiatric PGS prediction of verbal and nonverbal reasoning (see Figure S3 for the same results using psychiatric PGSs corrected for p). Overall, most disorders, except for ANX, ALCH, and OCD, showed at least 1 significant association with either verbal or nonverbal reasoning. Similar to what was observed for general cognitive ability, removing transdiagnostic genetic risk did not significantly alter psychiatric disorder–cognition associations (see Table S7).

However, when considering greater specificity at the level of cognitive skills, a clearer differentiation between the p-factor and disorder-specific effects began to emerge. The p-factor PGS exhibited a stronger association with nonverbal reasoning across multiple ages (β = −0.059, SE = 0.019, *p*_FDR_ = .018 at age 16 and β = −0.058, SE = 0.020, *p*_FDR_ = .021 at 23), while its association with verbal reasoning was only significant in early adulthood (β = −0.052, SE = 0.02, *p*_FDR_ = .046). In contrast, PGSs associated with individual disorders—including those for ADHD, ASD, AN, and BIP—displayed either stronger associations with verbal reasoning (e.g., AN and BIP) or significant links across all 3 cognitive domains across development (e.g., ADHD, autism, PTSD).

When we compared the strength of the PGS predictions for verbal and nonverbal reasoning, we found that ADHD was more strongly associated with nonverbal reasoning at ages 9 and 16, while BIP and AN were more significantly associated with verbal abilities (see Figure S2), although some of those differences did not survive FDR correction (Table S9). For other disorders, including ASD, ANX, depression, and SCZ, trends favoring either verbal or nonverbal abilities were observed, but the differences were not significant (Figure 2).

To further examine the apparent increase in associations between the ADHD and ASD PGSs and verbal and nonverbal cognitive abilities (Figure 2), we adopted the approach described above and compared the PGS prediction of a verbal/nonverbal common factor model with that of a specific effects model, where the PGS predicted the residual variance specific to each assessment not captured by the common factor. Consistent with the results obtained for general cognitive ability, the specific effects model provided a significantly better fit than the common factor model. This indicates that the observed pattern of developmental increase is more likely to reflect time-specific genetic associations rather than measurement instability (ADHD and verbal ability: χ^2^_5_ = 15.65, *p* = .008; ADHD and nonverbal ability: χ^2^_5_ = 21.80, *p* < .001; ASD and verbal ability: χ^2^_5_ = 16.19, *p* = .006). The only exception was for ASD and nonverbal cognitive ability, where the specific effects model showed a better, although not statistically significant, fit (χ^2^_5_ = 8.86, *p* = .11). When we constrained PGS effects to be equal across waves and compared this model with the one in which the effects were allowed to vary freely, the latter consistently showed a better fit. This suggests that the effects of PGSs on verbal and nonverbal cognitive ability become significantly stronger over developmental time (Tables S12 and S13).

Regarding spatial abilities, the p-factor was not associated with either the manipulation or navigation skills. Spatial manipulation ability was significantly predicted by the PGSs for SCZ, AN, ASD, and ADHD (Figure S4), although only the association between ADHD, ASD, and SCZ survived FDR correction (Table S5). Spatial navigation was significantly associated with genetic risk for ASD, PTSD, and SCZ, but only the association with SCZ remained significant after FDR correction (Table S5).

### Within-Family Analyses

We used within-family analyses to decompose the population-level PGS-cognition associations discussed so far into between- and within-family effects (52,55) (see Table S8). For some disorders (ADHD, ALCH, AN, and PTSD), PGS effects were mostly accounted for by between-family factors, while for others, associations remained significant also when looking at how differences between siblings in genetic risk for psychopathology predicted differences in cognition across development (Figures S5–S7 and S13).

**Figure 3:**
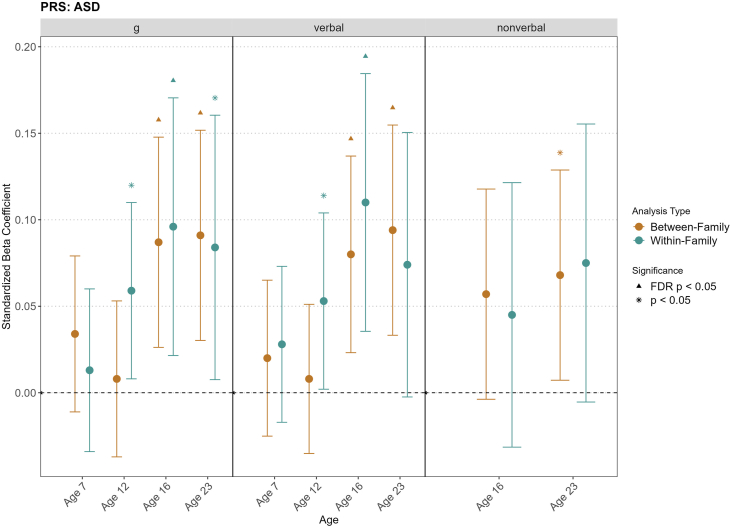
Family-level analyses of the association between ASD and general cognitive ability and verbal and nonverbal reasoning. This plot presents the within-family analyses for the significant associations identified for the ASD PRS. The brown dots represent between-family effects of the ASD polygenic scores on cognitive outcomes, and the green dots indicate within-family effects. The error bars indicate 95% CIs. The stars represent significance at a threshold of *p* < .05, and the triangles represent significance after FDR correction. ASD, autism spectrum disorder; FDR, false discovery rate; PRS, polygenic score.

Significant within-family PGS predictions were observed for ASD, BIP, SCZ, TS, and the p-factor (see Figures S9, S10, and S14; Table S8). ASD yielded the largest effects, with significant within-family effects observed at age 16 for general cognitive ability (β = 0.096, SE = 0.038, *p*_FDR_ = .038) (Figure 3) and verbal reasoning (β = 0.110, SE = 0.038, *p*_FDR_ = .025). BIP showed significant within-family effects at age 12 for verbal reasoning (β = 0.067, SE = 0.026, *p* = .010), SCZ showed significant within-family effects for spatial ability at age 19 (β = −0.161, SE = 0.064, *p* = .011), and TS showed a significant within-family effect at age 16 for general cognitive ability (β = −0.085, SE = 0.039, *p* = .029). Within-family effects of the p-factor on nonverbal reasoning were significant at age 16 (β = −0.085, SE = 0.04, *p* = .035). However, within-family effects did not survive FDR correction except for ASD.

ANX, depression, and OCD showed no significant within- or between-family level effects (Figures S8, S11, and S12). We also explored the associations between psychiatric PGSs and cognitive outcomes after accounting for transdiagnostic genetic effects. The results showed slight variations, but the overall pattern remained highly consistent (Table S8 and Figures S17–S24).

## Discussion

In this study, we examined the associations between genetic risks for psychopathology and cognitive development from early childhood to early adulthood. By leveraging PGSs derived from GWASs, we tested whether genetic risk for different psychiatric disorders, before and after we removed transdiagnostic effects, was associated with specific profiles of cognitive functioning over development. First, our results indicate that, contrary to what has been observed for the development of psychiatric symptoms, which are best predicted by transdiagnostic effects (56) the association between psychopathology and general cognitive ability is driven by disorder-specific genetic risk rather than transdiagnostic influences. Second, we found that genetic risk for different psychiatric disorders was differentially associated with specific cognitive profiles, with some disorders (e.g., BIP and AN) being more closely linked to verbal reasoning and others (e.g., ADHD) to nonverbal cognitive skills. Third, within-family analyses highlighted how these population-level associations capture a mixture of direct and indirect effects, with ASD-related genetic risk showing the most robust direct effects. These findings provide us with a deeper understanding of how genetic risk for psychiatric disorders is linked to the development of specific cognitive profiles and highlight how research into cognitive functioning in psychopathology would gain from focusing on individual disorders rather than transdiagnostic factors.

The first main finding of the study was that genetic risk linked to the transdiagnostic p-factor had limited predictive value for general cognitive abilities, with significant associations emerging only in early adulthood. In contrast, genetic vulnerability to individual psychiatric disorders predicted general cognitive ability both before and after controlling for the p-factor. These associations were consistently observed across development, and effect sizes increased over time. Sensitivity analyses indicated that the observed increase in associations is unlikely to be the product of measurement instability but more likely reflects increasing genetic associations between psychopathology and cognition from childhood to adulthood. However, disorder-cognition associations were not uniform across psychiatric conditions. For some conditions, such as ADHD, PTSD, and TS, associations were consistently negative, whereas for other disorders such as ASD, ALCH, AN, and BIP, they were consistently positive. This suggests that genetic risk related to some psychiatric conditions may confer potential cognitive advantages, while genetic risk associated with others may confer potential disadvantages.

Such divergent effects could be explained by pleiotropy, which may involve both biological and mediated pathways. Evidence for biological pleiotropy, where shared genetic factors influence both psychopathology and cognitive abilities, comes from well-replicated studies showing a shared genetic basis between several psychiatric conditions and cognition-related traits (17,55,57). Therefore, our findings may reflect this underlying shared genetic architecture. In addition, Mendelian randomization studies suggest potential causal relationships between psychiatric conditions and cognitive outcomes. For example, ADHD, MDD, and SCZ have been associated with lower educational attainment, whereas BIP has shown positive causal effects (58,59). However, mediating factors, such as pain and oxidative stress, have been proposed as alternative or additional pathways underlying these pleiotropic effects (58,59).

These results are consistent with previous findings on the association between psychiatric disorders and cognition. For example, ADHD has been extensively studied, with literature documenting that children and adults with ADHD achieve lower scores on cognitive tests across clinical (60,61) and subclinical (62,63) samples. A genetically informative study using a general population sample also found that polygenic risk for ADHD was negatively associated with performance IQ, even after controlling for a latent neurodevelopmental risk score (64). Deficits in cognitive functioning have also been associated with PTSD, beyond the effects of other psychiatric symptoms such as depression (65) and beyond the influence of traumatic experiences (66). In the case of TS, although findings have been less consistent (67), negative associations with general cognitive ability have been reported (68), especially in population-based samples (69).

On the other hand, for ASD, our results highlight its positive genetic correlation with general cognitive ability (17,57) and are consistent with a previous meta-analysis of the association between the ASD PGS and general cognitive ability across 3 independent cohorts (70). Although negative (71) or no associations between BIP and cognitive ability (72,73) have often been reported in the literature, it has been reported that genetic liability toward BIP is associated with skills related to cognitive outcomes such as creativity (74,75), educational attainment (76), and a lower risk of cognitive deficits (77). Genetic liability to AN was positively associated with cognitive performance (17), and our findings are consistent with research that does not consider genetic risk (78).

These results suggest that cognitive characteristics related to psychopathology differ for different psychiatric disorders and that disorder-specific genetic factors play a more substantial role in shaping cognitive outcomes than transdiagnostic effects. Therefore, rather than focusing on investigating relationships between transdiagnostic genetic risk and cognition (13,79, 80, 81), studies would benefit from investigating these relationships separately for each psychiatric disorder. This contrasts with findings on genetic vulnerability to psychiatric symptoms, where transdiagnostic genetic effects provide the strongest predictive power across both diagnostic categories and symptom continua (56,82). Our findings highlight the complexity of psychiatric disorders and their associated differences in cognitive functioning.

Our results also revealed nuanced associations between psychiatric disorders and cognitive domains. For example, many disorders demonstrated robust predictive effects on specific cognitive abilities, with the ADHD PGS showing the strongest and developmentally increasing negative association with nonverbal reasoning. In contrast, the AN and BIP PGSs were more positively associated with verbal abilities across development. In terms of spatial cognitive ability, only ADHD and SCZ were negatively associated with spatial manipulation abilities. These results reflect previous clinical findings that individuals with ADHD perform significantly lower in spatial and nonverbal abilities compared with healthy control individuals, including domains such as visuospatial perception and abstract thinking (83), working memory (84), and processing speed (85), where inattention may contribute to poorer performance on tasks with a higher cognitive load. A meta-analysis of 26 studies also showed that patients with ADHD had greater deficits in visuospatial storage and manipulation skills compared with verbal reasoning (86). On the other hand, genetic vulnerability to ASD was found to be equally linked to verbal and nonverbal reasoning. Consistent with our findings, previous studies have found that verbal and nonverbal reasoning contributed equally to language outcomes in children with ASD (87) and that adults with ASD demonstrated homogeneous cognitive profiles for verbal and nonverbal reasoning, irrespective of the severity of their ASD symptoms (88).

At the family level, we found that the pathways through which genetic risk for psychiatric disorders contribute to cognitive development vary across disorders. For ADHD, ALCH, AN, PTSD, and TS, the effects were primarily operating through between-family pathways. In contrast, ASD, BIP, and SCZ exhibited both within- and between-family effects. This is consistent with previous research that found evidence for direct genetic effects on neurodevelopmental symptoms in early childhood (20,21). This distinction underscores the heterogeneity in how different disorders contribute to cognitive outcomes and highlights the importance of disentangling different genetic and environmental pathways developmentally (55,89,90). It also suggests that disorders may be differentially influenced by individual- and population-level factors; therefore, interventions aimed at mitigating the impact of psychopathology on cognitive functioning, and vice versa, should consider these different exposures as well as specific vulnerability.

Several limitations of the current study should be acknowledged. First, although TEDS is largely representative of the population of England and Wales for their birth cohort, it is not representative of the current population. For example, the TEDS families scored higher on socioeconomic outcomes compared with the current population mean. This is particularly important as socioeconomic outcomes have been linked to cognitive development (91) and mental health (92), and it limits the generalizability of our findings to broader populations and new generations of children. Second, while our study suggests that the observed increase in the association between psychopathology PGSs and cognitive functioning is likely due to increasing genetic links rather than measurement instability, we did not directly model development using a longitudinal framework, given measurement heterogeneity. Future studies that directly model longitudinal trajectories, such as latent growth models, may illuminate the dynamic relations between cognitive functioning and symptoms of psychopathology. Third, our cognitive measures, while comprehensive, may not fully capture the multidimensional nature of cognitive development (93). Incorporating additional specific measures such as tests of executive functioning and memory could provide a more nuanced picture (94). Fourth, it should also be acknowledged that PGS effects were small, particularly when decomposing associations into between- and within-family associations. Fifth, we acknowledge that while we modeled transdiagnostic effects using a common factor model, the p-factor has been modeled differently, for example using a hierarchical factor model (12,18). However, this is unlikely to have substantially affected the main findings, as transdiagnostic effects were found to play only a marginal role in their association with cognitive development.

## Supplementary Material

Supplemental Note and Figures S1–S24

Tables S1–S13

Key Resource Table
