# Supplementary material for: Disorder-Specific Genetic Effects Drive the Associations Between Psychopathology and Cognitive Functioning
Source: Biol Psychiatry Glob Open Sci. 2025 Dec 18;6(2):100680. doi: 10.1016/j.bpsgos.2025.100680 (PMC12870865; doi:10.1016/j.bpsgos.2025.100680)
Supplement: Supplemental Note and Figures S1–S24 [file mmc1.pdf]

SUPPLEMENTARY INFORMATION

Disorder-Specific Genetic Effects Drive the Associations Between Psychopathology and Cognitive Functioning

Liao *et al.*

List of Contents

Sup. Note Pre-registered cognitive change score analysis……………………………………………………..3
Figure S1. Significant differences in cognitive ability prediction of psychiatry disorders before and after controlling for the p factor. 4
Figure S2. Verbal and Non-Verbal Cognitive Ability Comparison. 5
Figure S3. Verbal and Nonverbal Cognitive Ability with Psychiatric Polygenic Scores after correcting for p factor. 6
Figure S4. Spatial Cognitive Ability with Psychiatric Polygenic Scores before and after correcting for p factor. 7
Figure S5. Direct and Indirect Genetic Effects of genetic risk of ADHD on Cognitive Abilities…….. 8
Figure S6. Direct and Indirect Genetic Effects of genetic risk of ALCH on Cognitive Abilities………9
Figure S7. Direct and Indirect Genetic Effects of genetic risk of AN on Cognitive Abilities. ……..... 10
Figure S8. Direct and Indirect Genetic Effects of genetic risk of ANX on Cognitive Abilities. ………11
Figure S9. Direct and Indirect Genetic Effects of genetic risk of ASD on Cognitive Abilities. ………12
Figure S10. Direct and Indirect Genetic Effects of genetic risk of BIP on Cognitive Abilities. ……. 13
Figure S11. Direct and Indirect Genetic Effects of genetic risk of MDD on Cognitive Abilities…... 14
Figure S12. Direct and Indirect Genetic Effects of genetic risk of OCD on Cognitive Abilities……. 15
Figure S13. Direct and Indirect Genetic Effects of genetic risk of PTSD on Cognitive Abilities….. 16
Figure S14. Direct and Indirect Genetic Effects of genetic risk of SCZ on Cognitive Abilities…….17
Figure S15. Direct and Indirect Genetic Effects of genetic risk of TS on Cognitive Abilities. ……...18
Figure S16. Direct and Indirect Genetic Effects of genetic risk of p factor on Cognitive Abilities. 19
Figure S17. Direct and Indirect Genetic Effects of genetic risk of ADHD non-p on Cognitive Abilities. 20
Figure S18. Direct and Indirect Genetic Effects of genetic risk of ALCH non-p on Cognitive Abilities. 21
Figure S19. Direct and Indirect Genetic Effects of genetic risk of AN non-p on Cognitive Abilities. 22
Figure S20. Direct and Indirect Genetic Effects of genetic risk of ASD non-p on Cognitive Abilities. 23
Figure S21. Direct and Indirect Genetic Effects of genetic risk of BIP non-p on Cognitive Abilities. 24
Figure S22. Direct and Indirect Genetic Effects of genetic risk of PTSD non-p on Cognitive Abilities. 25
Figure S23. Direct and Indirect Genetic Effects of genetic risk of SCZ non-p on Cognitive Abilities. 26
Figure S24. Direct and Indirect Genetic Effects of genetic risk of TS non-p on Cognitive Abilities. 27

Supplementary Note: Pre-registered cognitive change score analysis

This supplementary note outlines the pre-registered analysis conducted to examine cognitive change scores and their association with genetic risk for psychopathology. The objective was to investigate how genetic predispositions to psychiatric conditions relate to cognitive development from childhood to adulthood.

Using the composite cognitive measures described in the main text, we calculated cognitive change scores for general cognitive ability, verbal, and nonverbal reasoning. These scores represented an individual's relative cognitive development from childhood to adulthood within the cohort. Specifically, cognitive change scores were computed by subtracting standardized cognitive scores at age 7 from standardized scores at age 21. Participants with change scores more than one standard deviation (SD) below the cohort mean were classified as having slower cognitive development, whereas those with scores more than one SD above the mean were classified as having faster development relative to their peers. Sample sizes for each group across the three domains are presented in Supplementary Table 1.

Participants were divided into two groups—higher cognitive change and lower cognitive change—based on their scores being ≥1 SD above or below the cohort mean. Regression analyses between the genetic risks of psychopathology and cognitive change scores were conducted separately for each group to test whether the relationship between polygenic risk scores for various psychiatric disorders and cognitive change scores varied by developmental trajectory.

The regression results are shown in Supplementary Table 6. In summary, the analyses revealed limited significant associations, which could be due to small sample size in each group. For general cognitive ability, polygenic scores for obsessive-compulsive disorder and Tourette's syndrome were significantly associated with cognitive change only in the faster development group. In contrast, verbal reasoning change scores were predicted by depression and Tourette's polygenic scores in the slower development group, and by PTSD polygenic scores in the faster development group. No polygenic scores significantly predicted nonverbal reasoning changes in either group. However, none of these associations survived false discovery rate (FDR) correction.

Figure S1: Significant differences in cognitive ability prediction of psychiatry disorders before and after controlling for the transdiagnostic p factor.

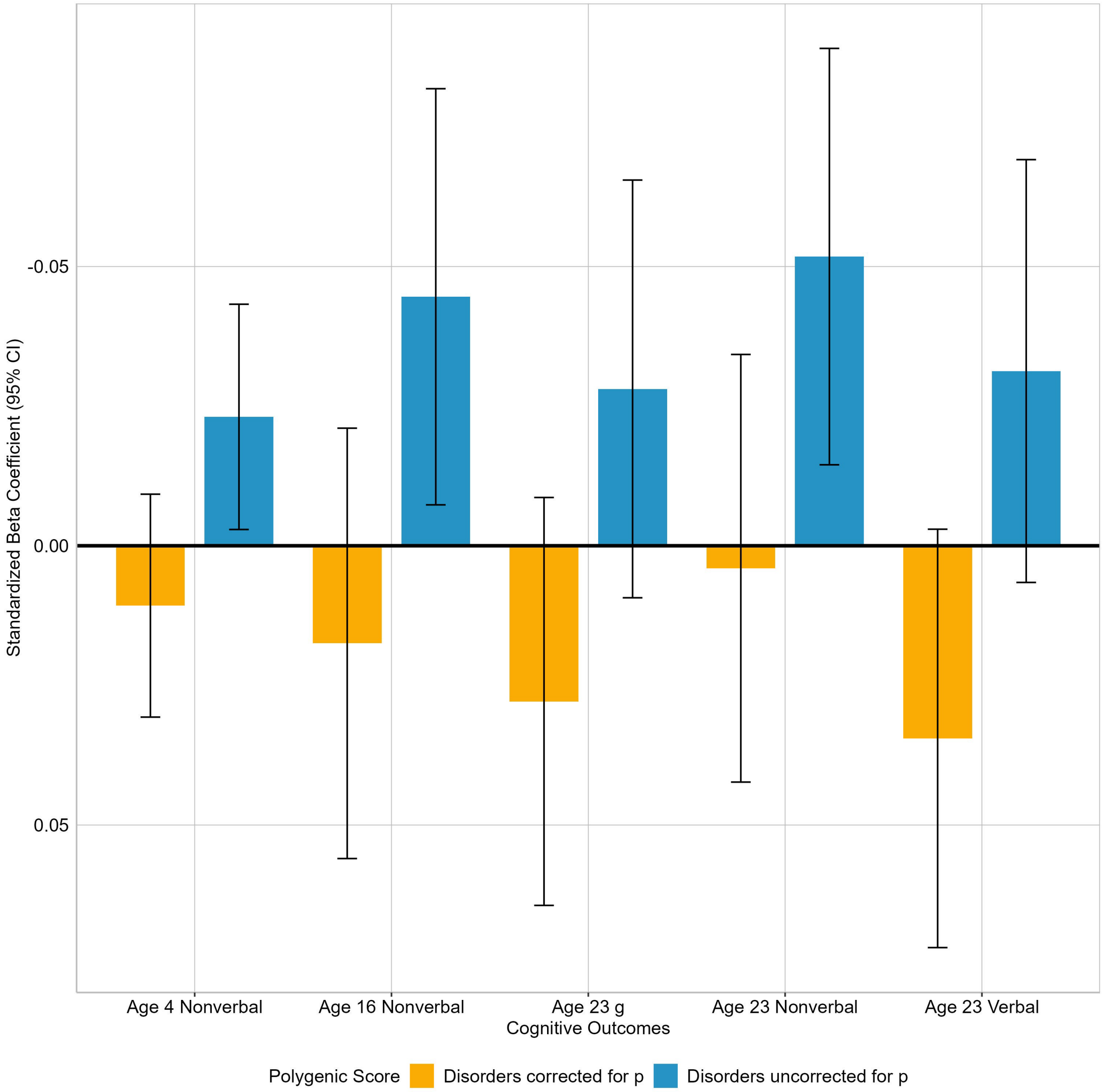

The figure shows the significant differences in cognitive ability prediction of psychiatry disorders before and after controlling for the transdiagnostic p factor. The differences are statistically significant at the 0.05 level.

Figure S2: Significant Differences in Associations Between Verbal and Non-Verbal Cognitive Ability and Psychiatric Polygenic Scores.

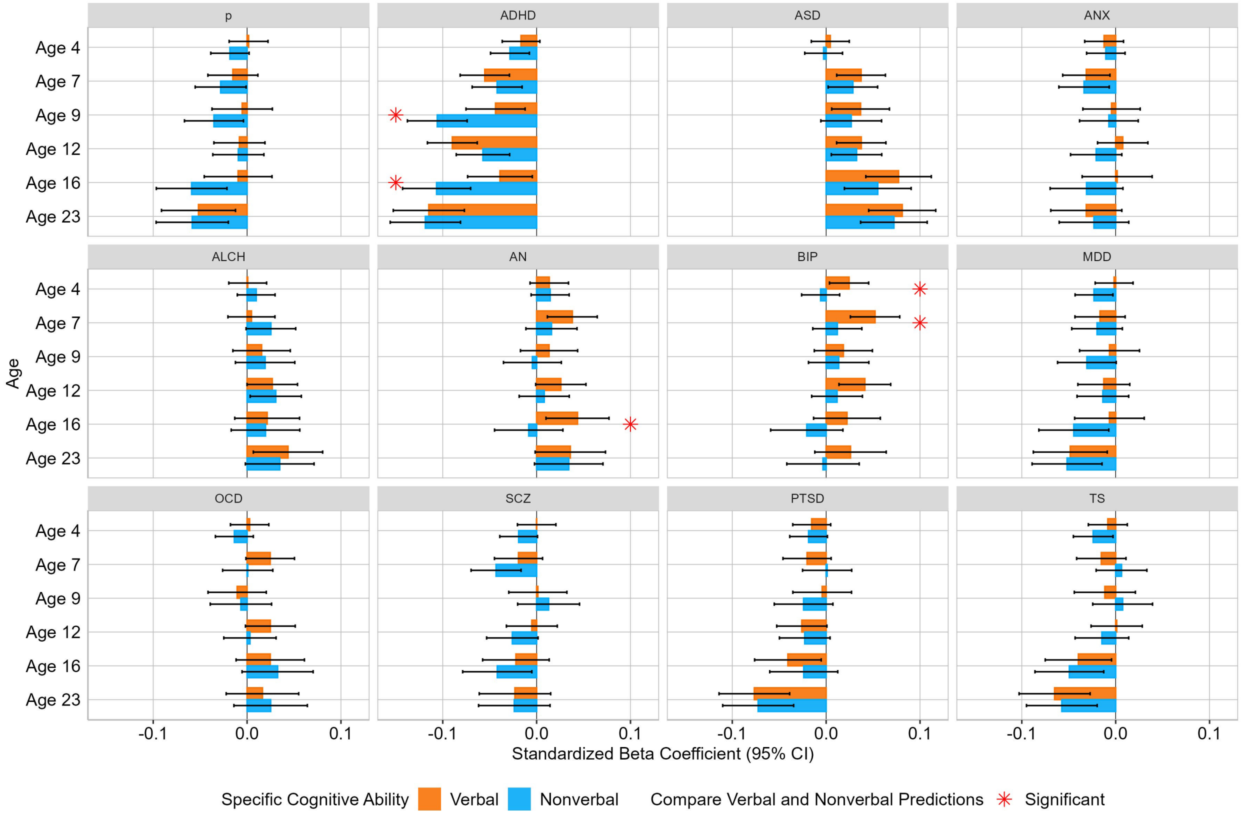

The figure shows the comparison between verbal and non-verbal cognitive ability for each psychiatric disorder PGS uncorrected for p factor. The differences are statistically significant at the 0.05 level.

Figure S3: Associations Between Verbal and Non-Verbal Cognitive Ability and Psychiatric Polygenic Scores after Correcting for p factor.

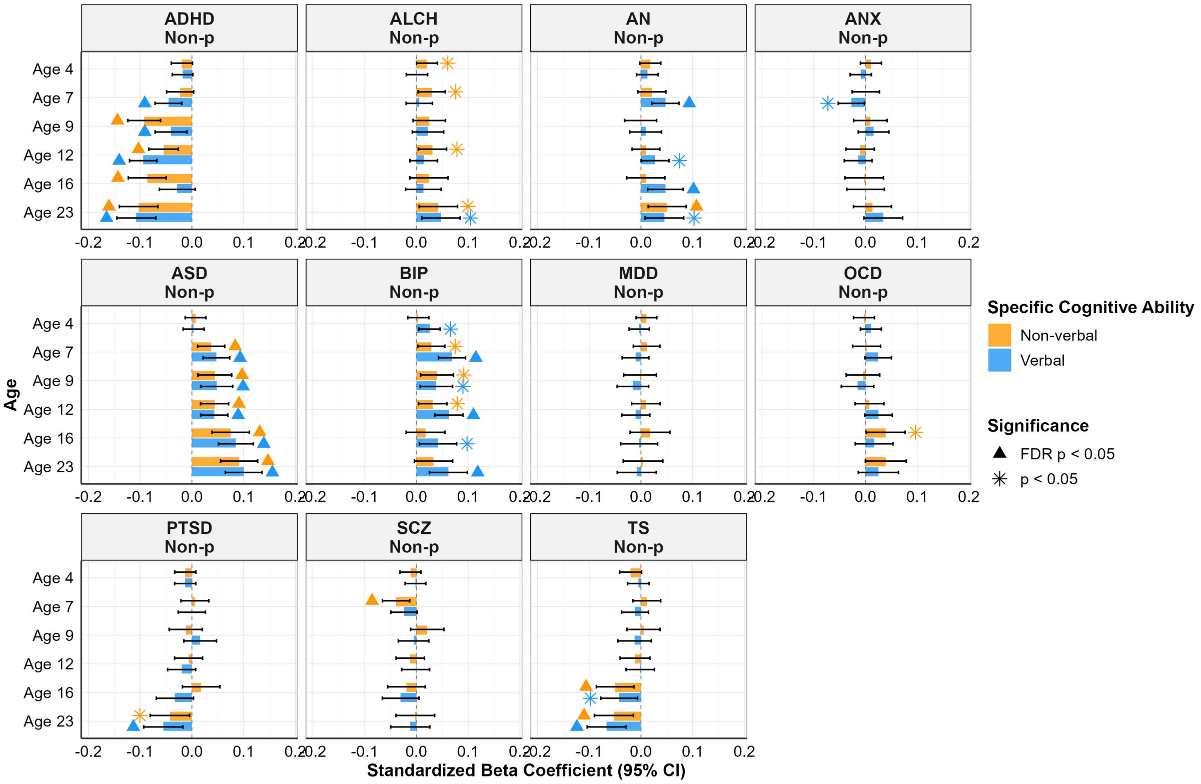

The figure shows the associations between specific cognitive ability and psychiatric polygenic scores after correcting for p factor.

Figure S4: Spatial Cognitive Ability with Psychiatric Polygenic Scores before and after correcting for p factor.

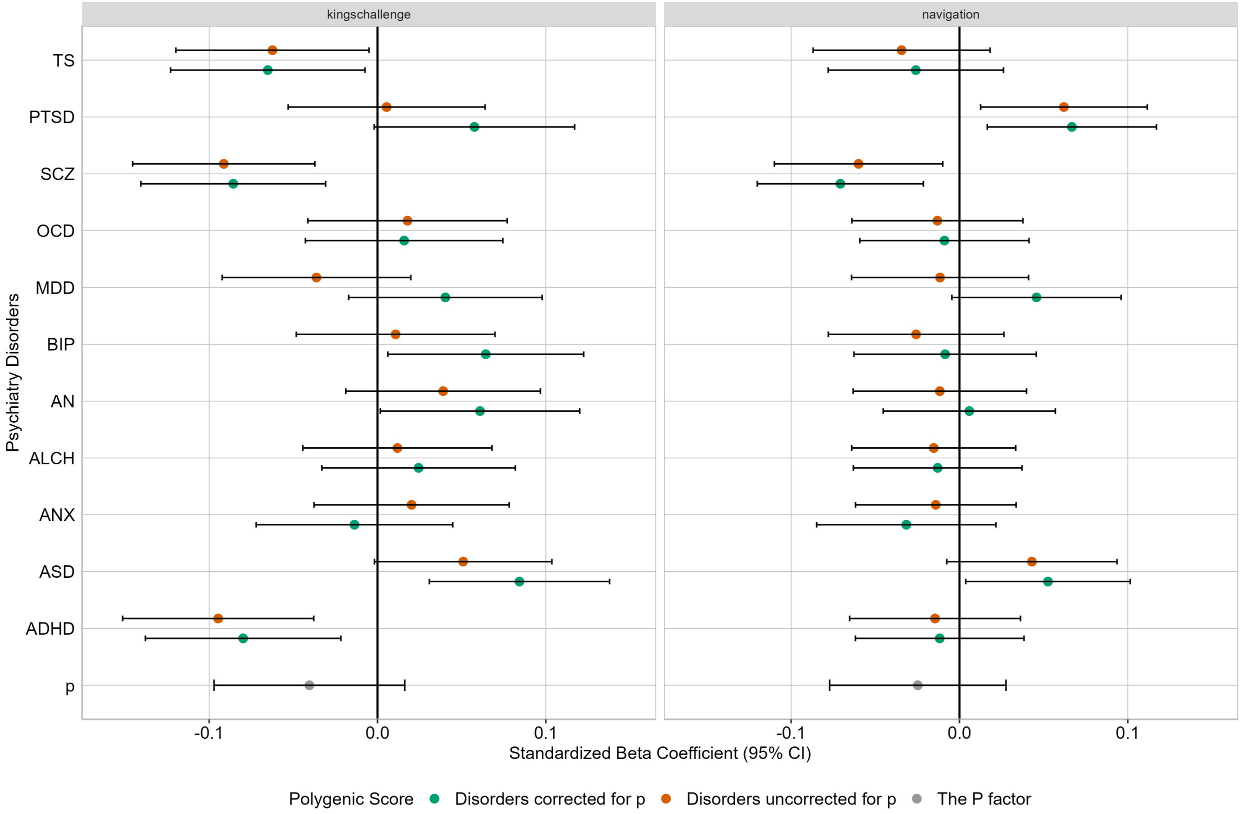

The figure illustrates spatial cognitive abilities and their associations with psychiatric polygenic scores, comparing results before and after correcting for the transdiagnostic p factor.

Figure S5: Direct and Indirect Genetic Effects of genetic risk of ADHD on Cognitive Abilities.

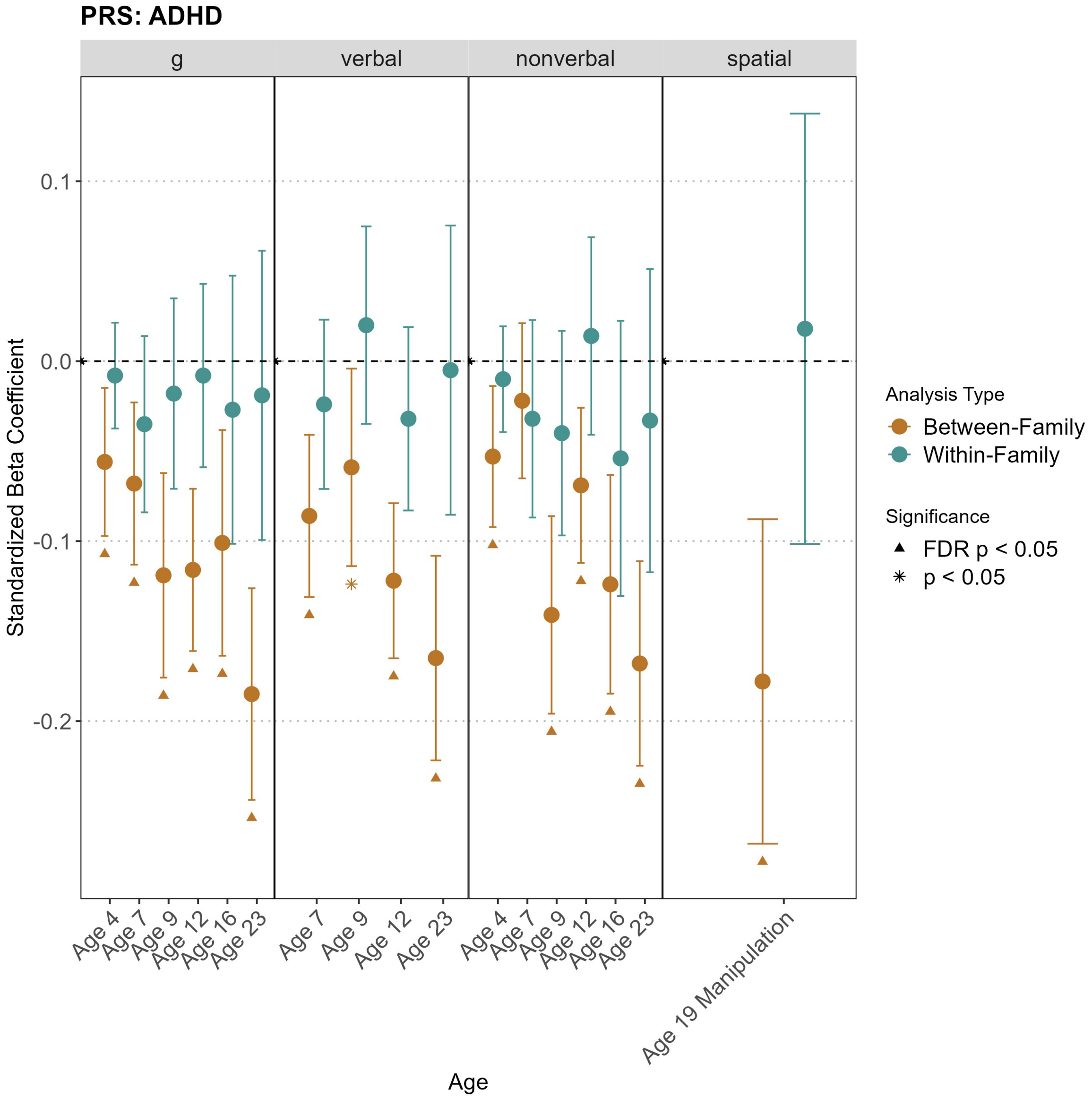

The figure presents the direct genetic effects and family-mediated indirect genetic effects of ADHD polygenic scores on cognitive abilities across development.

Figure S6: Direct and Indirect Genetic Effects of genetic risk of ALCH on Cognitive Abilities.

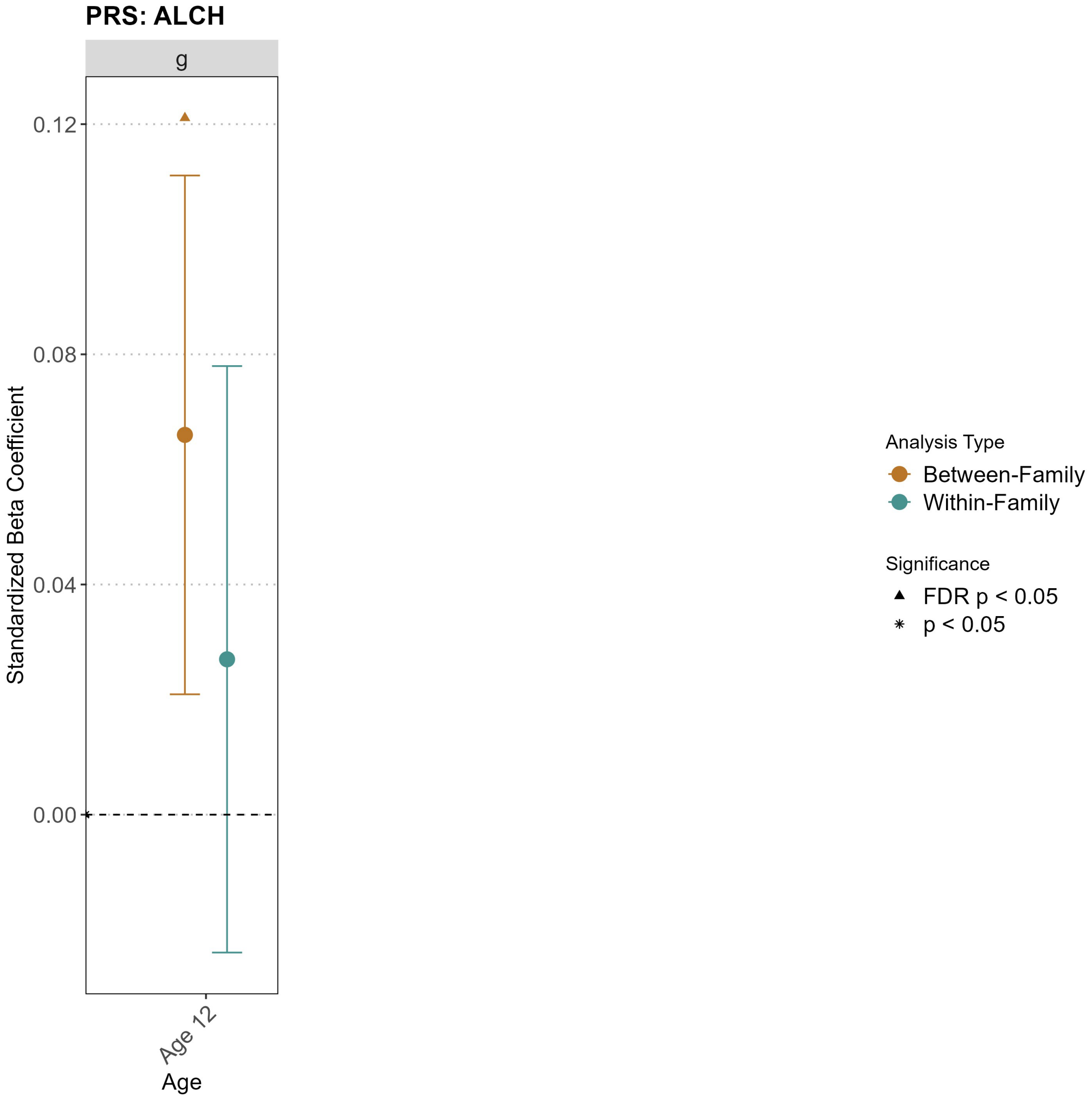

The figure presents the direct genetic effects and family-mediated indirect genetic effects of alcohol use disorder polygenic scores on cognitive abilities across development.

Figure S7: Direct and Indirect Genetic Effects of genetic risk of AN on Cognitive Abilities.

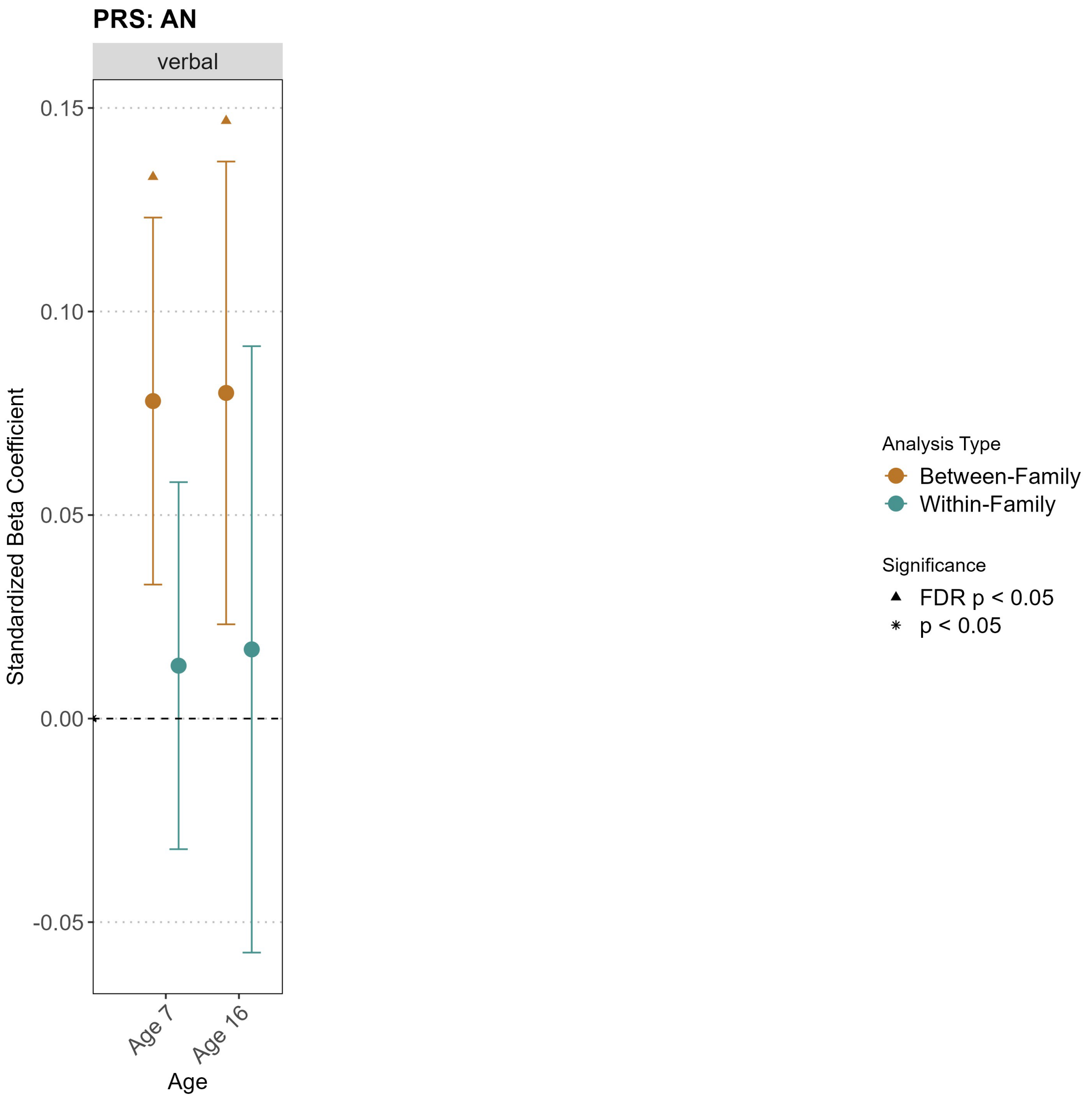

The figure presents the direct genetic effects and family-mediated indirect genetic effects of anorexia nervosa polygenic scores on cognitive abilities across development.

Figure S8: Direct and Indirect Genetic Effects of genetic risk of ANX on Cognitive Abilities.

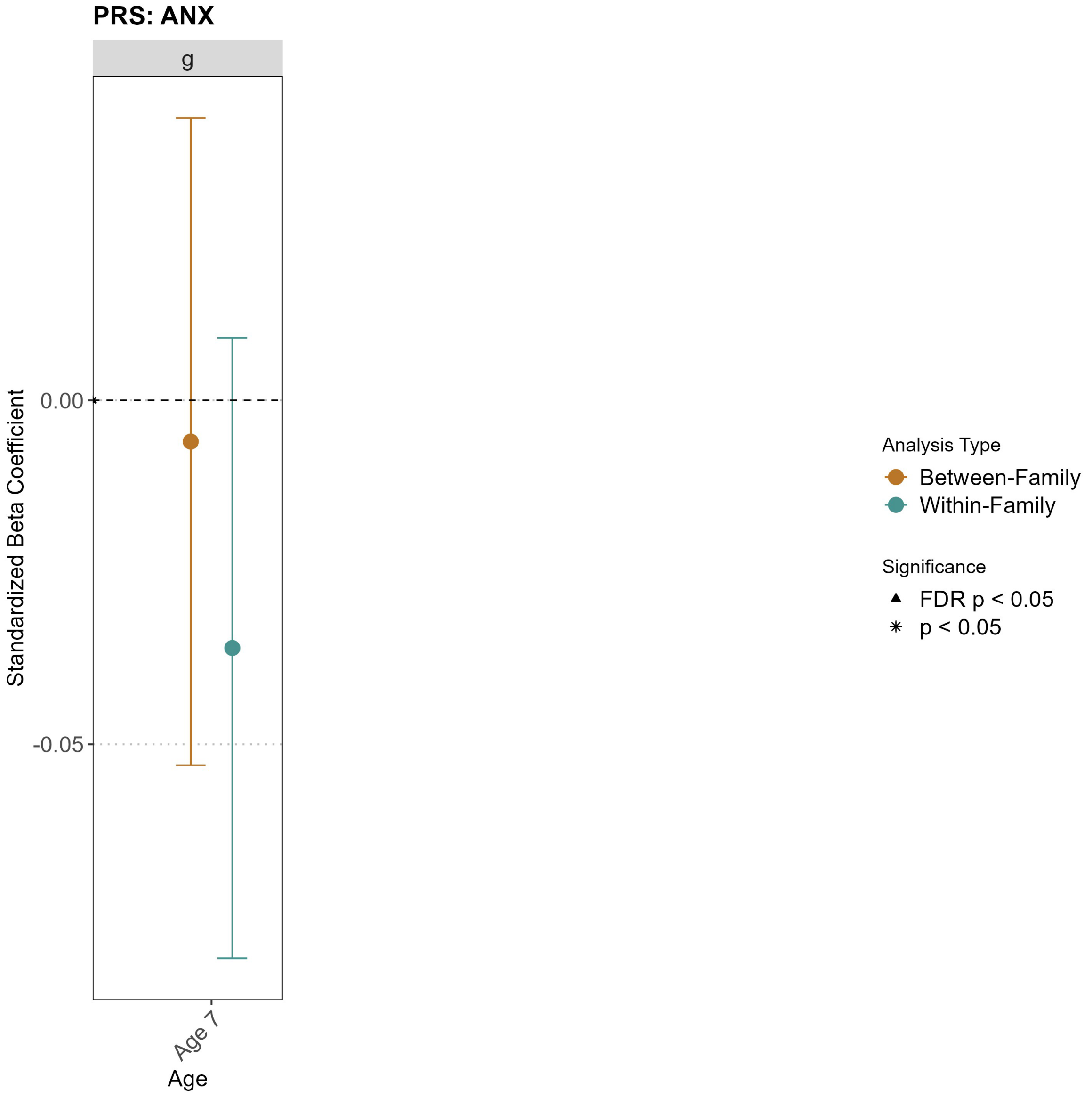

The figure presents the direct genetic effects and family-mediated indirect genetic effects of anxiety disorder polygenic scores on cognitive abilities across development.

Figure S9: Direct and Indirect Genetic Effects of genetic risk of ASD on Cognitive Abilities.

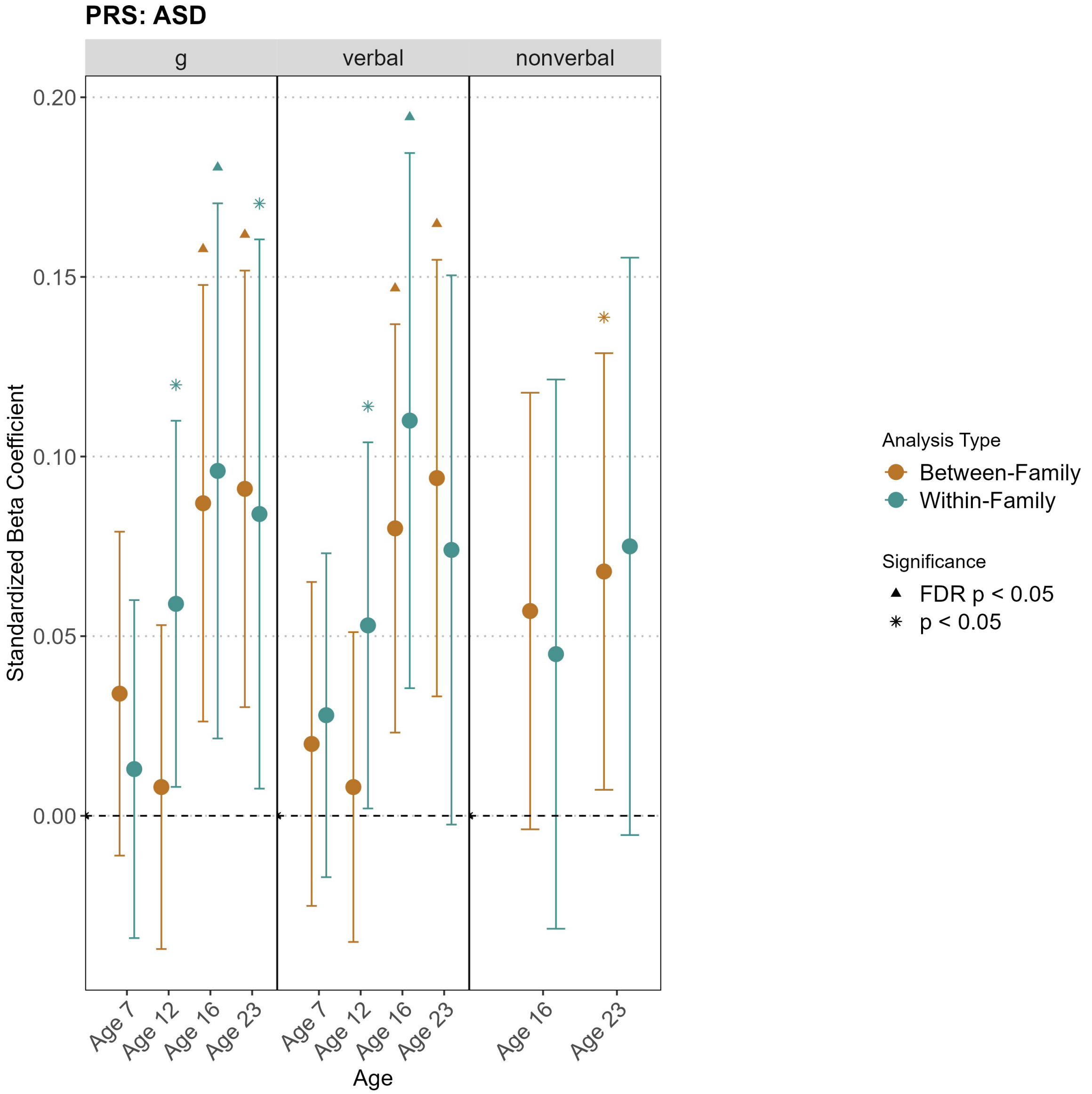

The figure presents the direct genetic effects and family-mediated indirect genetic effects of autism spectrum disorder polygenic scores on cognitive abilities across development.

Figure S10: Direct and Indirect Genetic Effects of genetic risk of BIP on Cognitive Abilities.

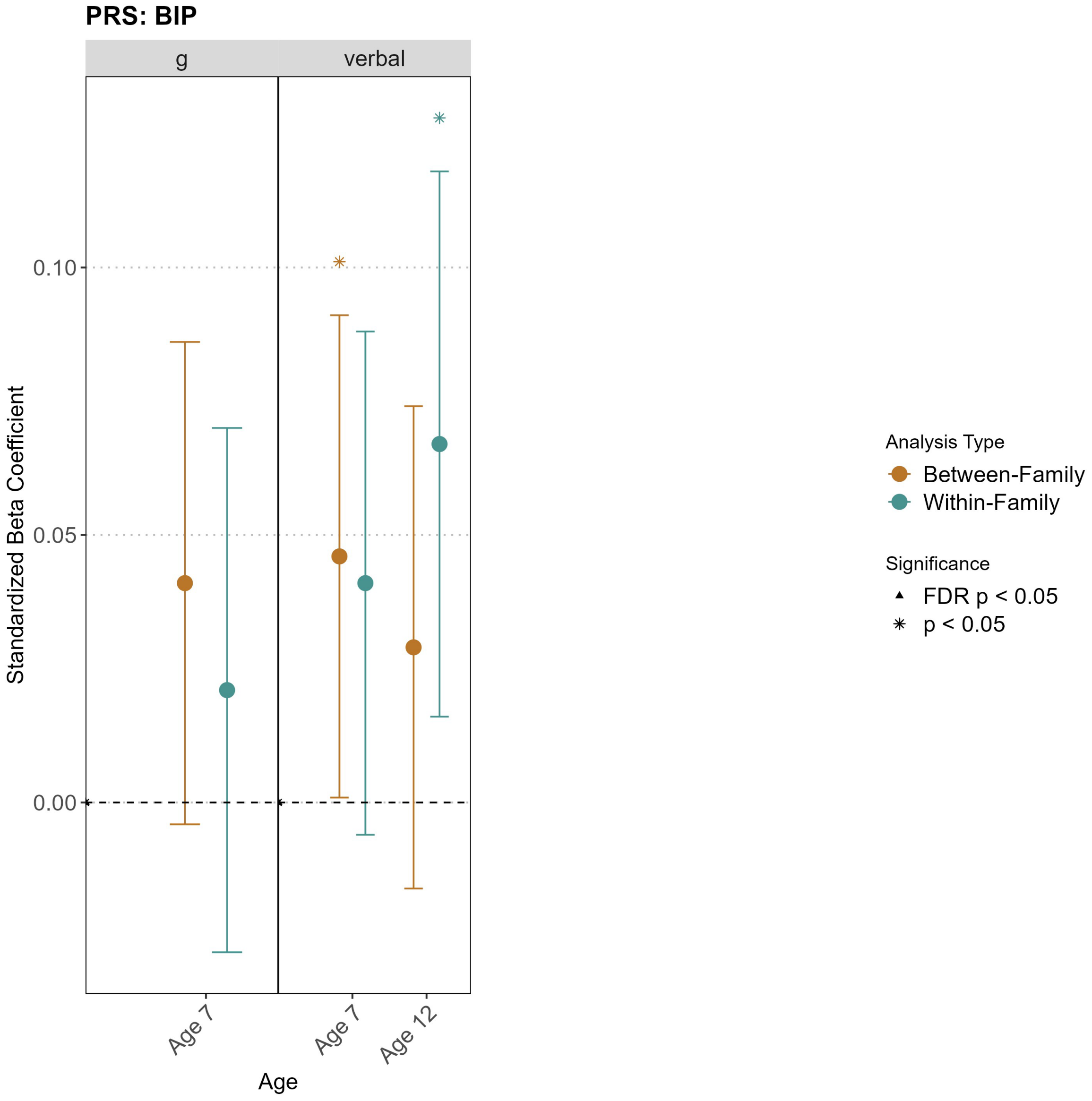

The figure presents the direct genetic effects and family-mediated indirect genetic effects of bipolar disorder polygenic scores on cognitive abilities across development.

Figure S11: Direct and Indirect Genetic Effects of genetic risk of MDD on Cognitive Abilities.

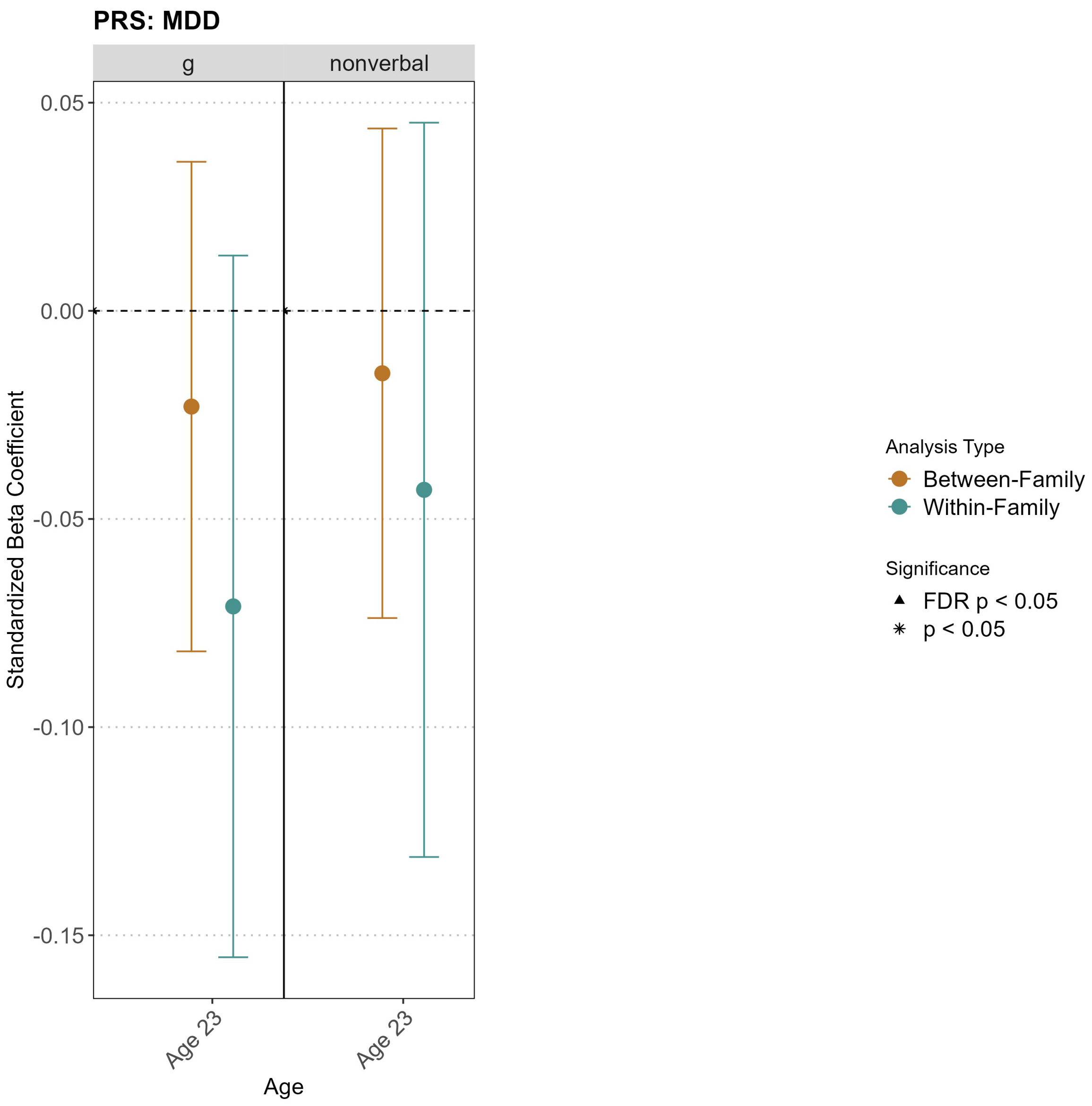

The figure presents the direct genetic effects and family-mediated indirect genetic effects of major depressive disorder polygenic scores on cognitive abilities across development.

Figure S12: Direct and Indirect Genetic Effects of genetic risk of OCD on Cognitive Abilities.

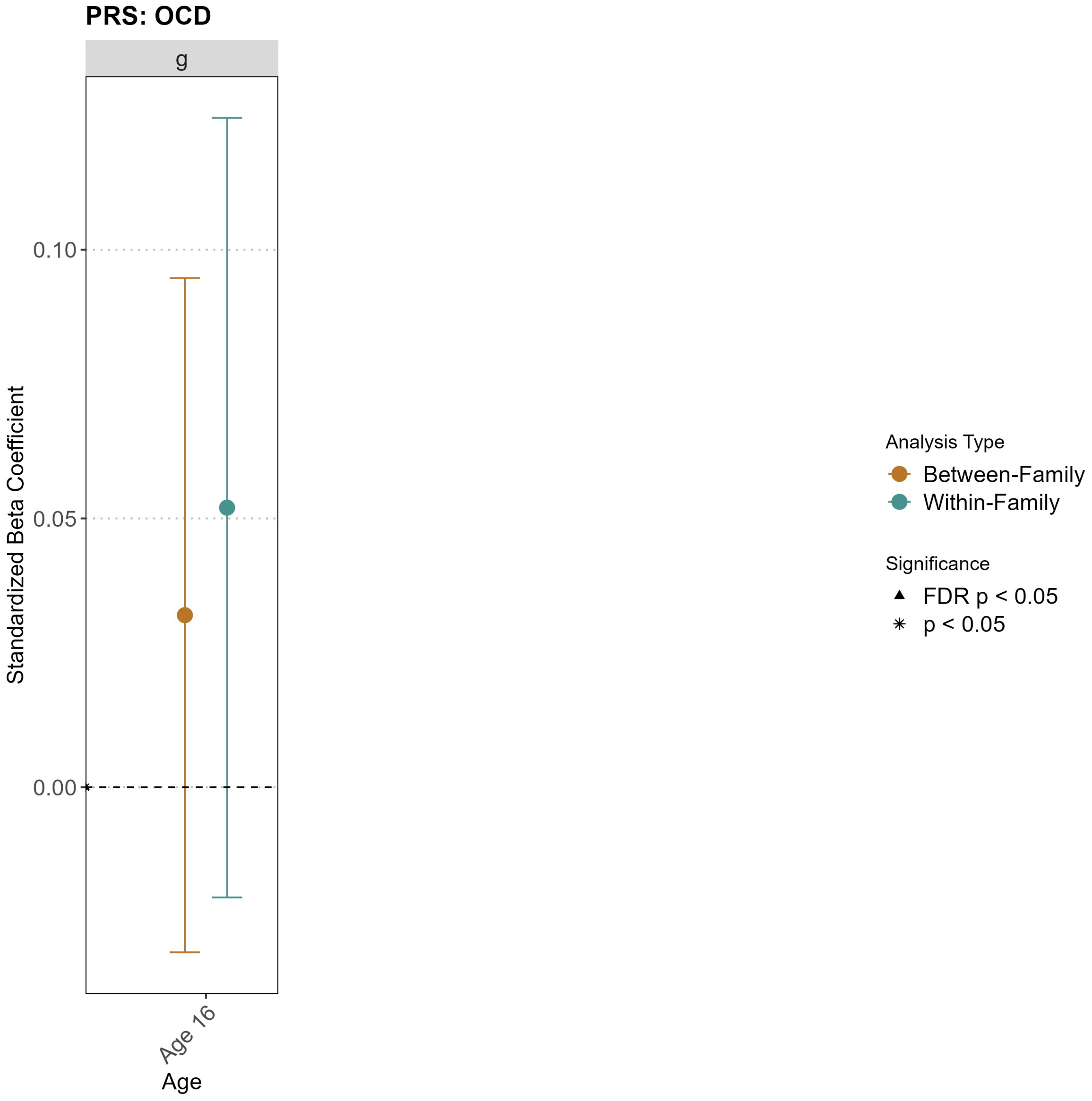

The figure presents the direct genetic effects and family-mediated indirect genetic effects of obsessive-compulsive disorder polygenic scores on cognitive abilities across development.

Figure S13: Direct and Indirect Genetic Effects of genetic risk of PTSD on Cognitive Abilities.

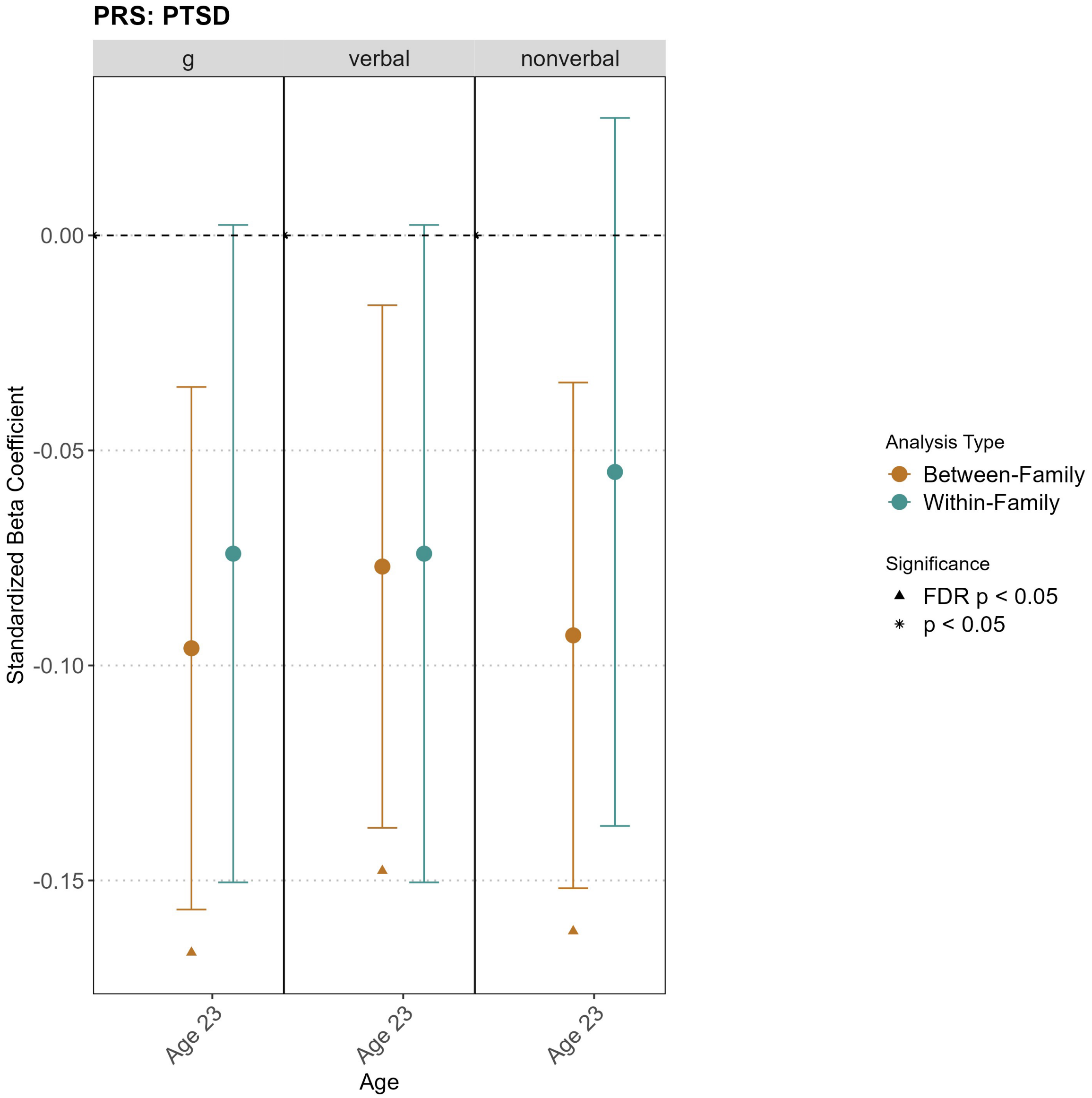

The figure presents the direct genetic effects and family-mediated indirect genetic effects of post-traumatic stress disorder polygenic scores on cognitive abilities across development.

Figure S14: Direct and Indirect Genetic Effects of genetic risk of SCZ on Cognitive Abilities.

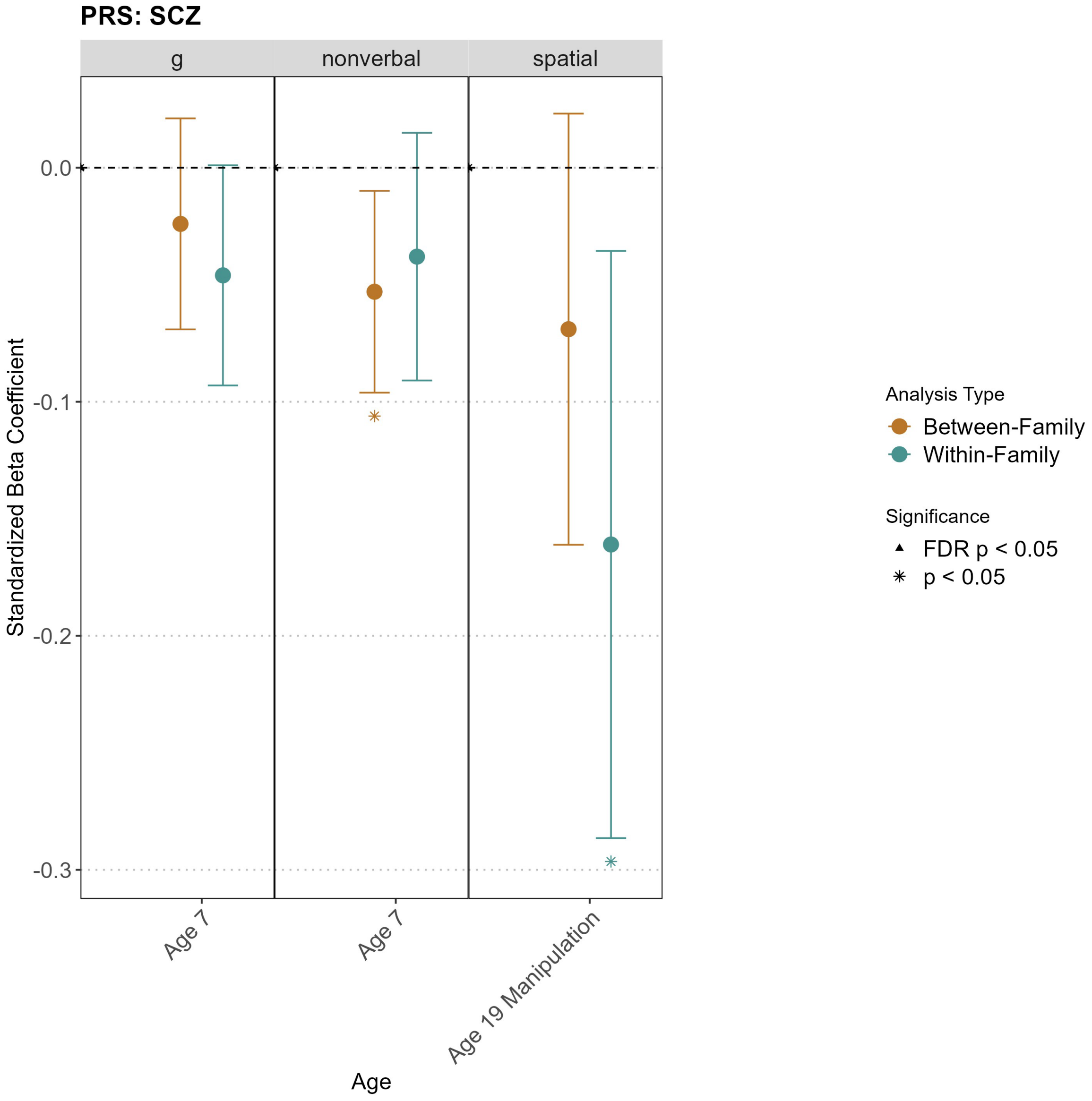

The figure presents the direct genetic effects and family-mediated indirect genetic effects of schizophrenia polygenic scores on cognitive abilities across development.

Figure S15: Direct and Indirect Genetic Effects of genetic risk of TS on Cognitive Abilities.

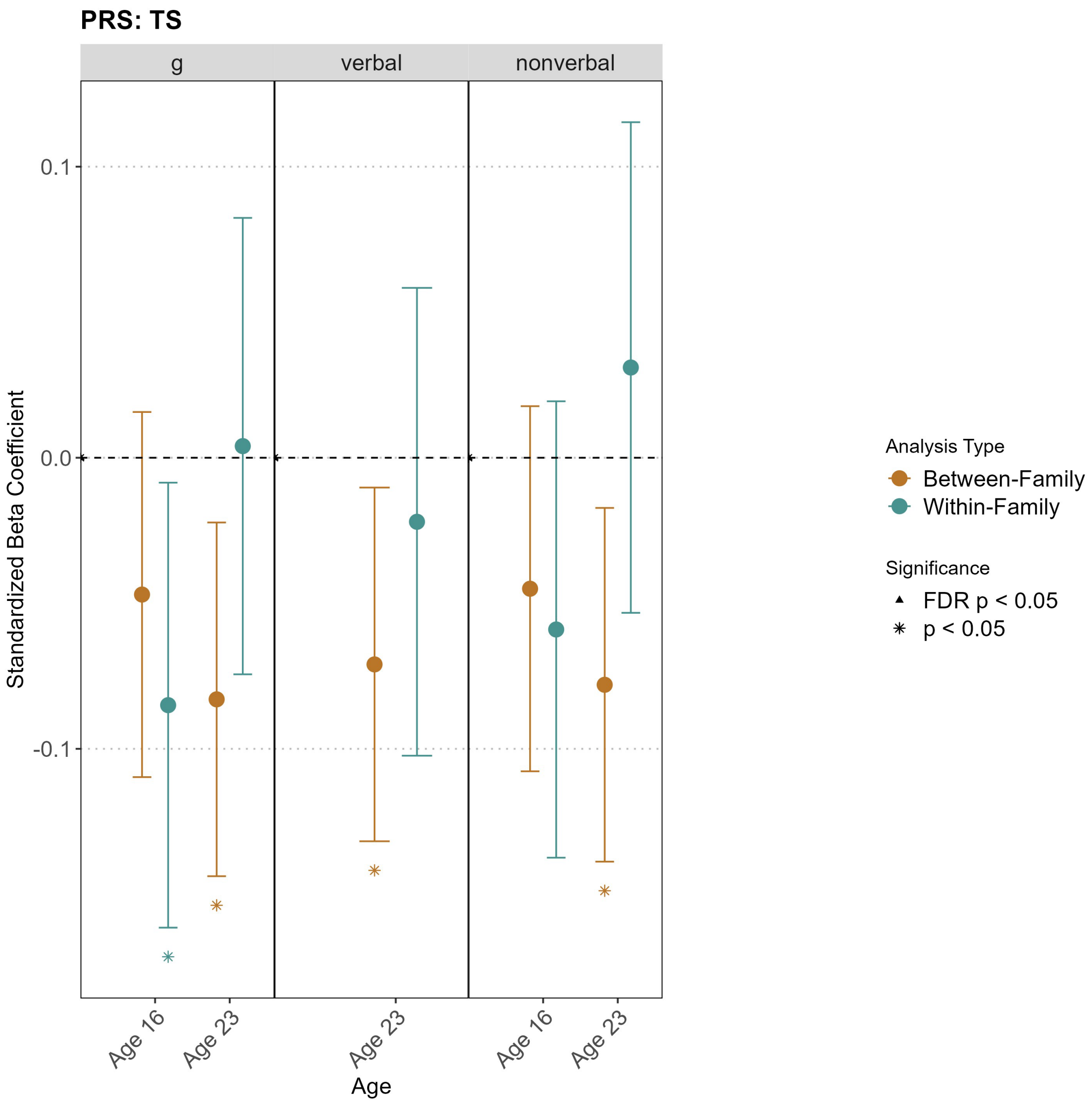

The figure presents the direct genetic effects and family-mediated indirect genetic effects of Tourette syndrome polygenic scores on cognitive abilities across development.

Figure S16: Direct and Indirect Genetic Effects of genetic risk of p factor on Cognitive Abilities.

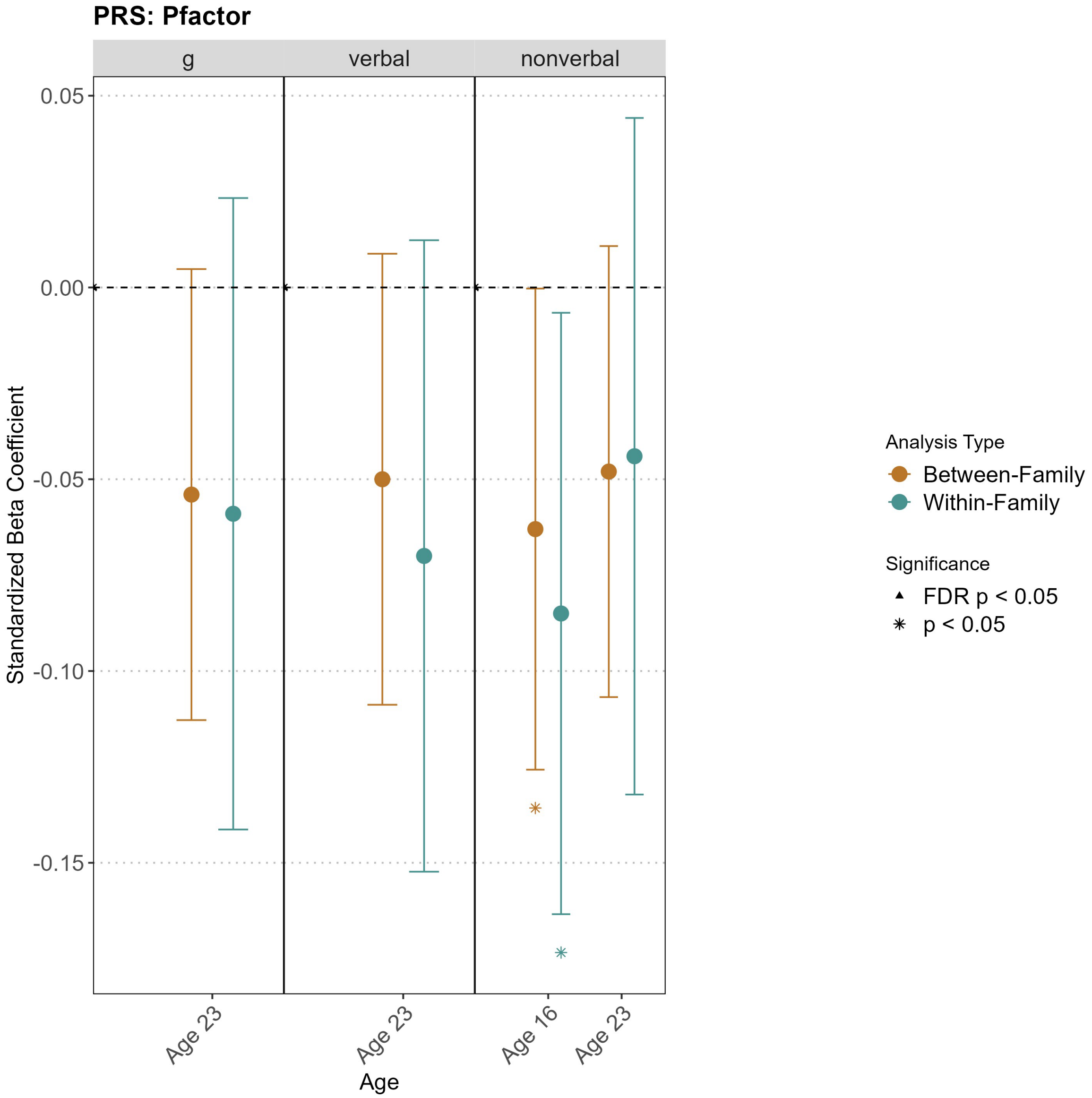

The figure presents the direct genetic effects and family-mediated indirect genetic effects of transdiagnostic p factor polygenic scores on cognitive abilities across development.

Figure S17: Direct and Indirect Genetic Effects of genetic risk of ADHD non-p on Cognitive Abilities.

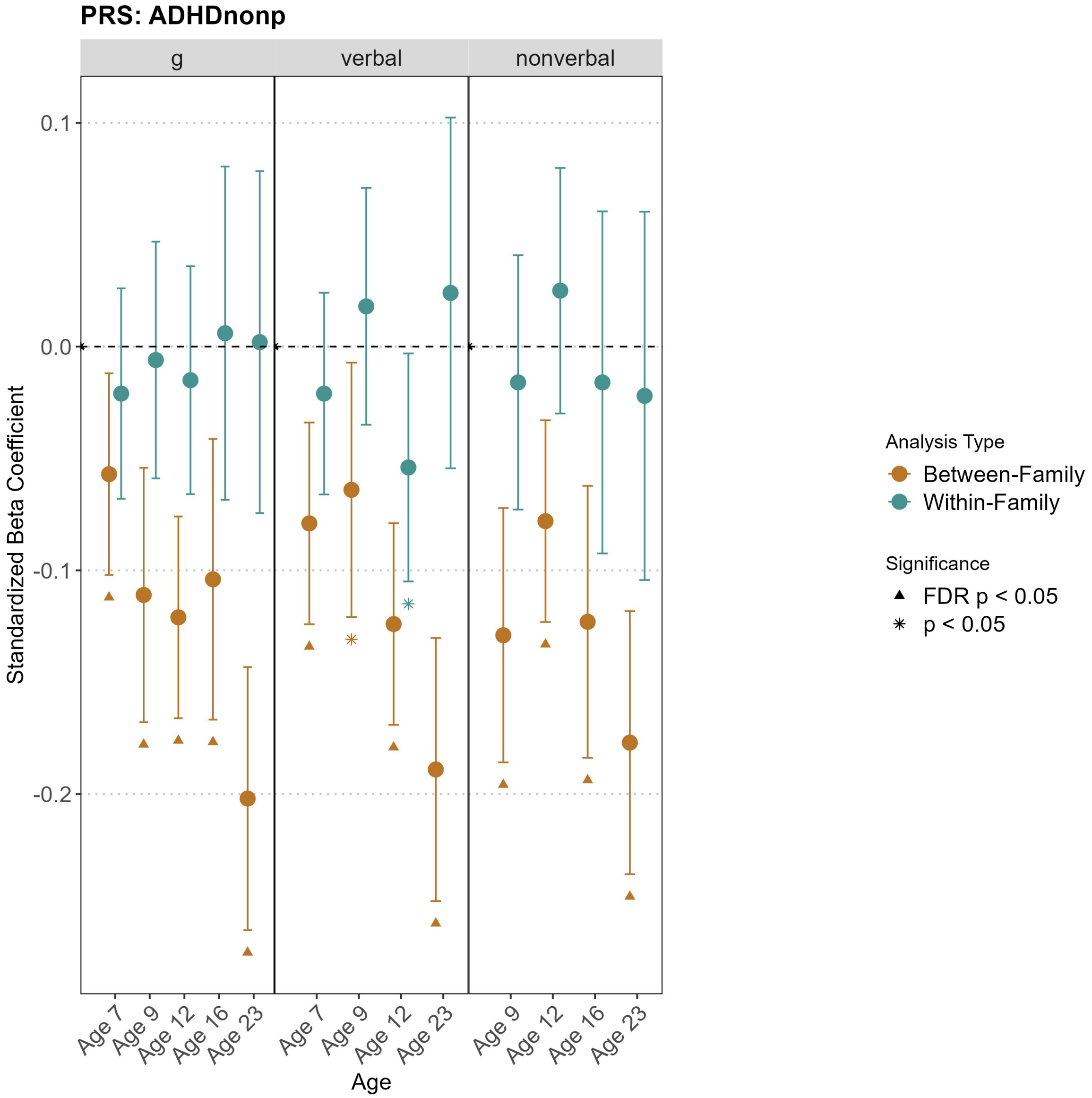

The figure presents the direct genetic effects and family-mediated indirect genetic effects of ADHD polygenic scores (corrected for p factor) on cognitive abilities across development.

Figure S18: Direct and Indirect Genetic Effects of genetic risk of ALCH non-p on Cognitive Abilities.

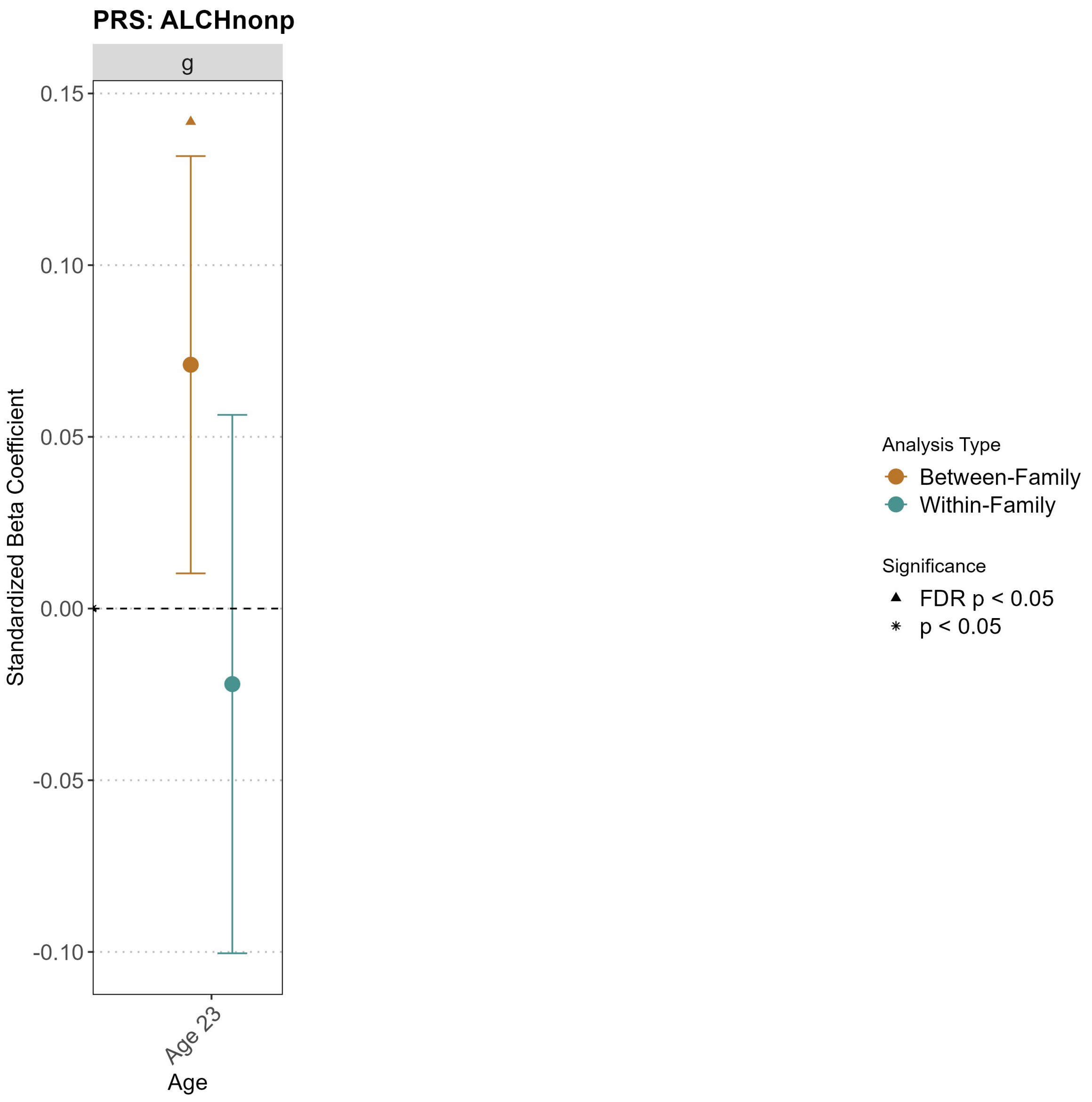

The figure presents the direct genetic effects and family-mediated indirect genetic effects of alcohol use disorder polygenic scores (corrected for p factor) on cognitive abilities across development.

Figure S19: Direct and Indirect Genetic Effects of genetic risk of AN non-p on Cognitive Abilities.

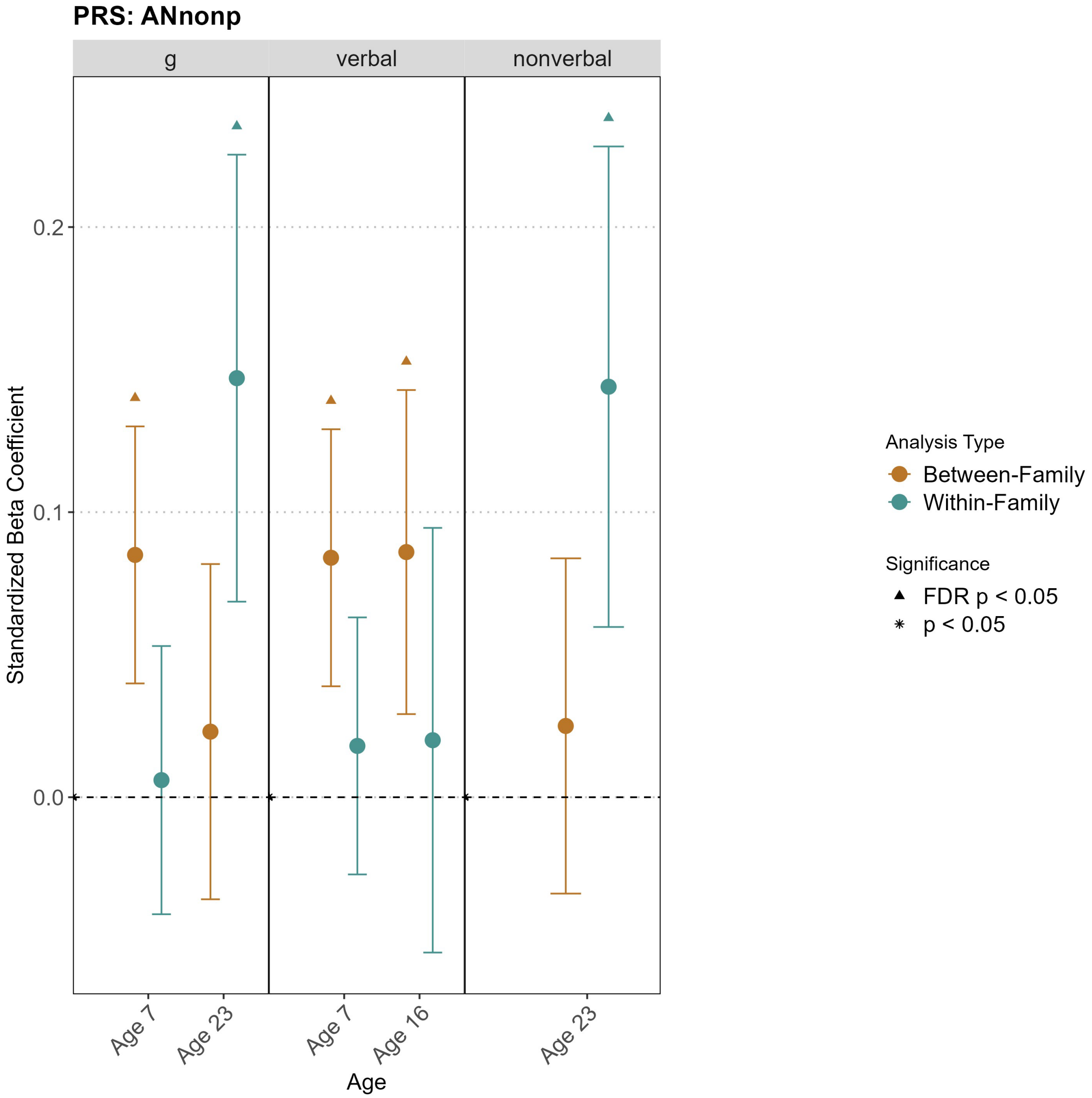

The figure presents the direct genetic effects and family-mediated indirect genetic effects of anorexia nervosa polygenic scores (corrected for p factor) on cognitive abilities across development.

Figure S20: Direct and Indirect Genetic Effects of genetic risk of ASD non-p on Cognitive Abilities.

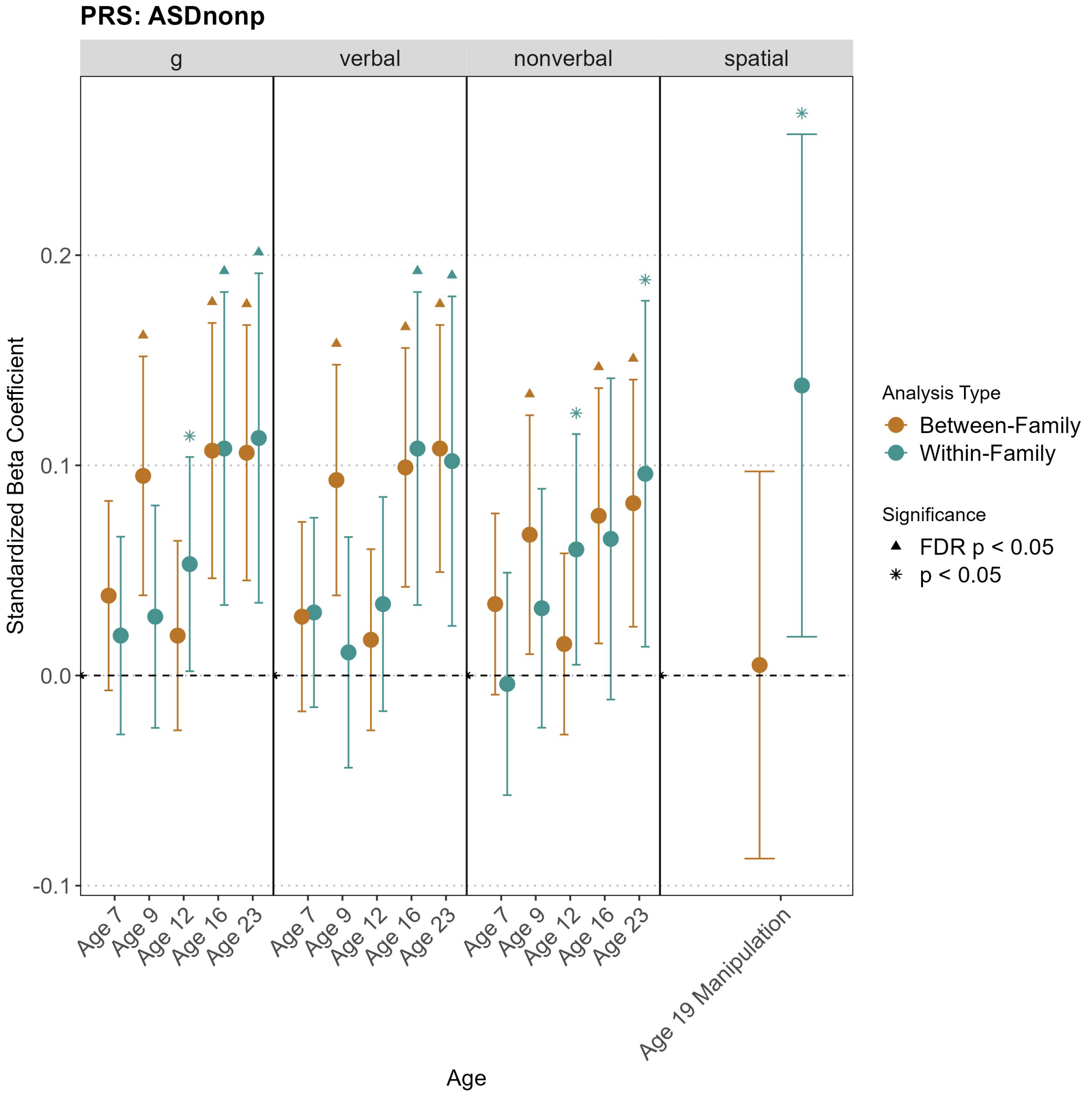

The figure presents the direct genetic effects and family-mediated indirect genetic effects of autism spectrum disorder polygenic scores (corrected for p factor) on cognitive abilities across development.

Figure S21: Direct and Indirect Genetic Effects of genetic risk of BIP non-p on Cognitive Abilities.

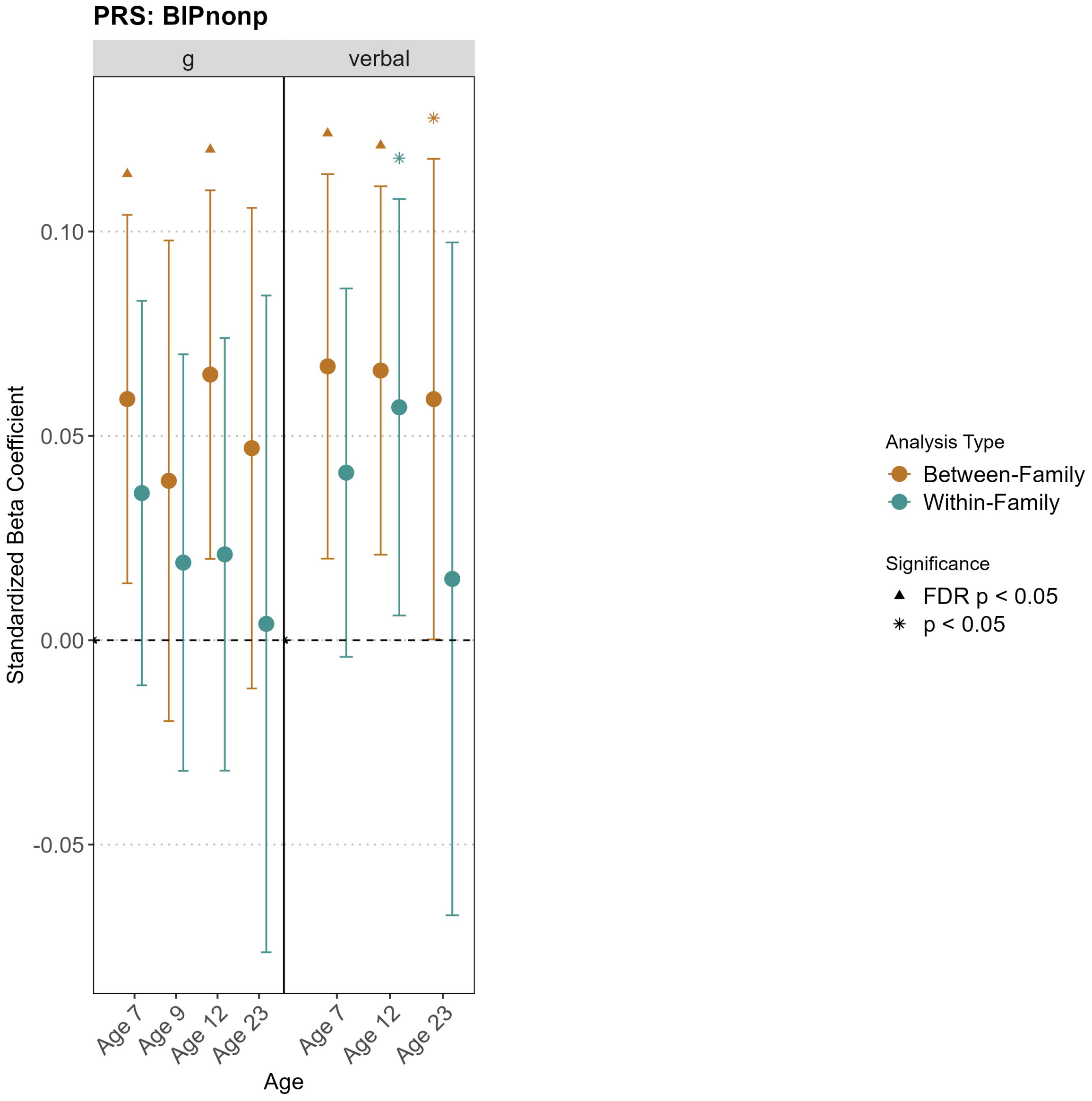

The figure presents the direct genetic effects and family-mediated indirect genetic effects of bipolar disorder polygenic scores (corrected for p factor) on cognitive abilities across development.

Figure S22: Direct and Indirect Genetic Effects of genetic risk of PTSD non-p on Cognitive Abilities.

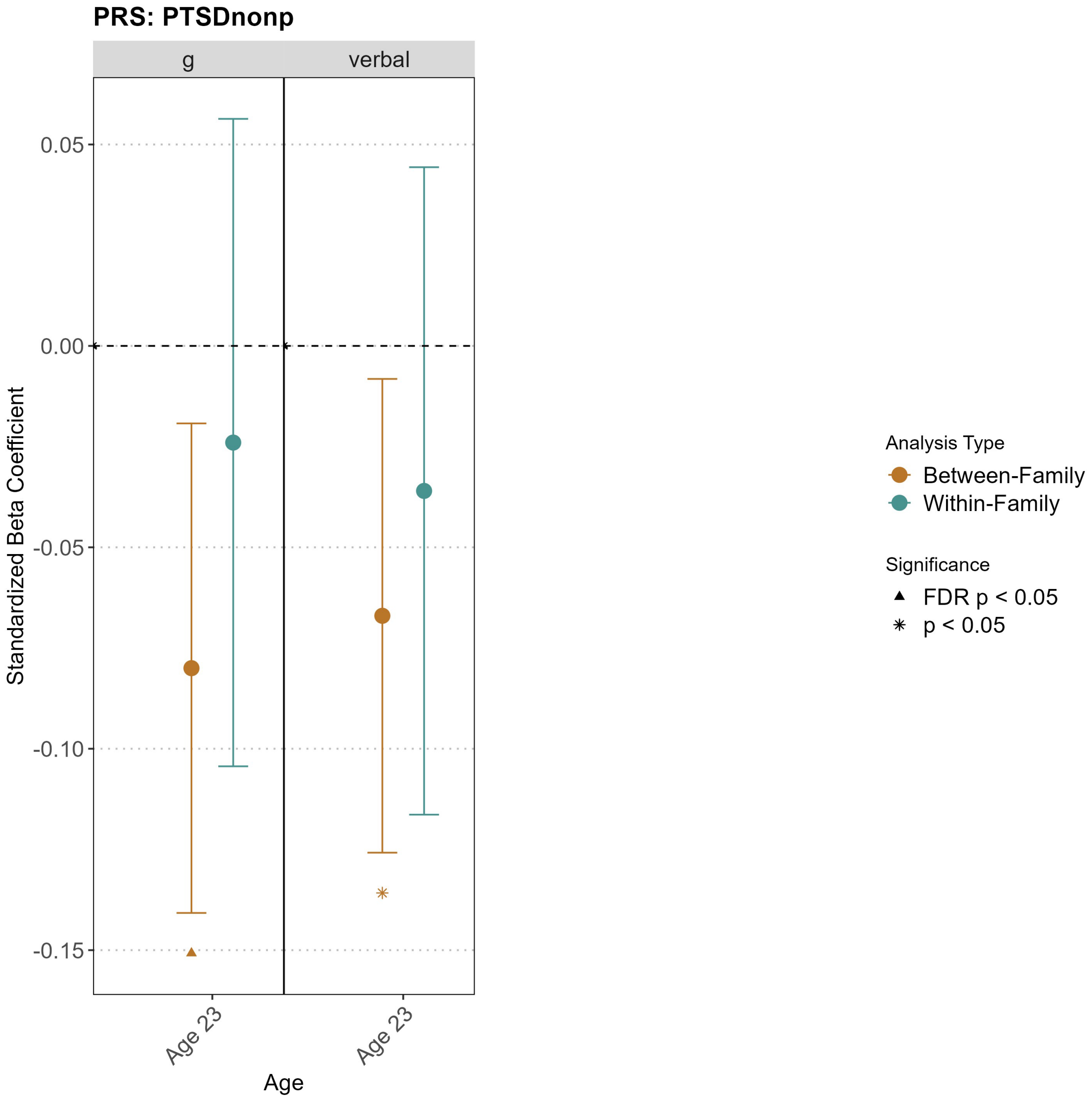

The figure presents the direct genetic effects and family-mediated indirect genetic effects of post-traumatic stress disorder polygenic scores (corrected for p factor) on cognitive abilities across development.

Figure S23: Direct and Indirect Genetic Effects of genetic risk of SCZ non-p on Cognitive Abilities.

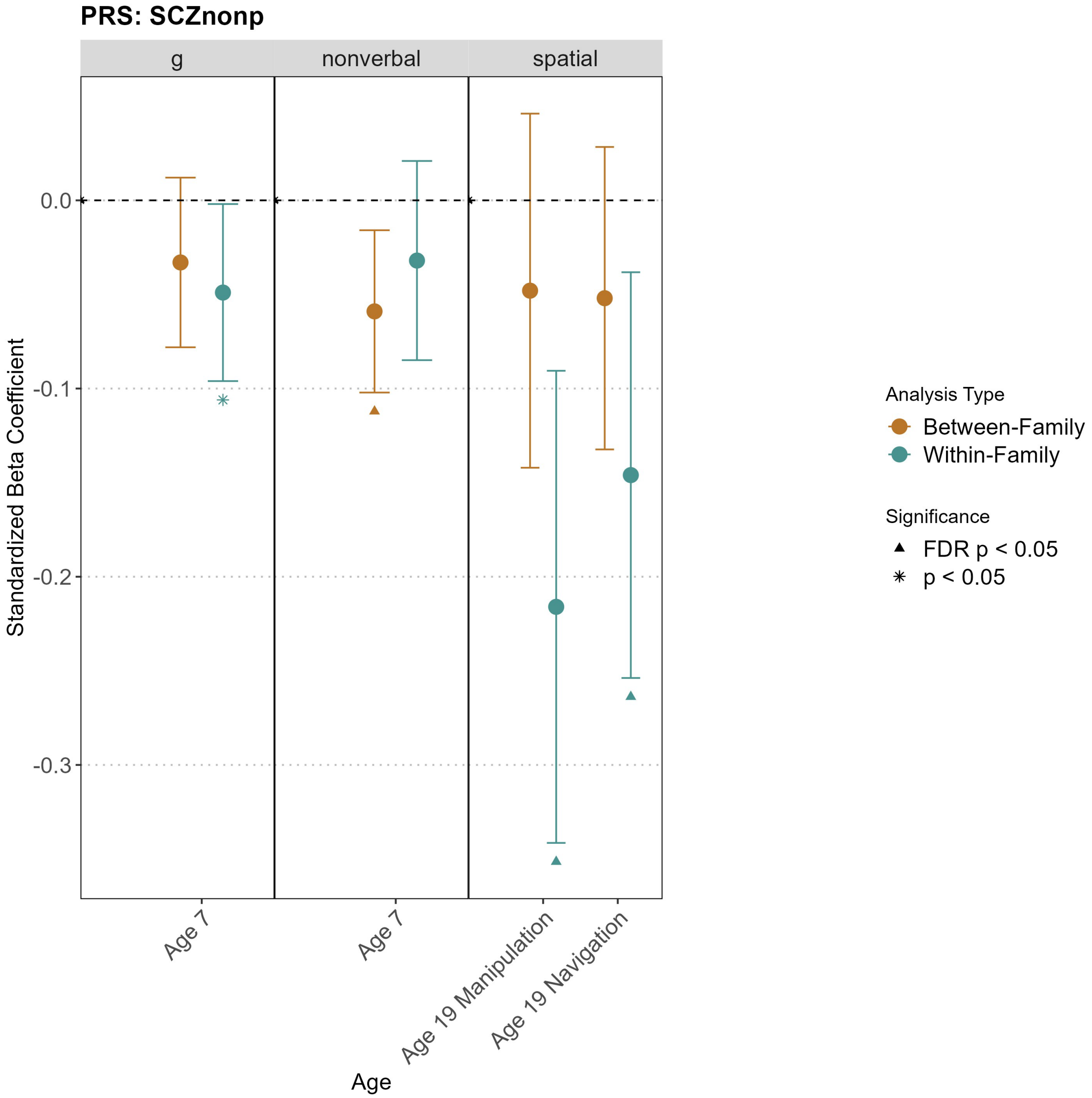

The figure presents the direct genetic effects and family-mediated indirect genetic effects of schizophrenia polygenic scores (corrected for p factor) on cognitive abilities across development.

Figure S24: Direct and Indirect Genetic Effects of genetic risk of TS non-p on Cognitive Abilities.

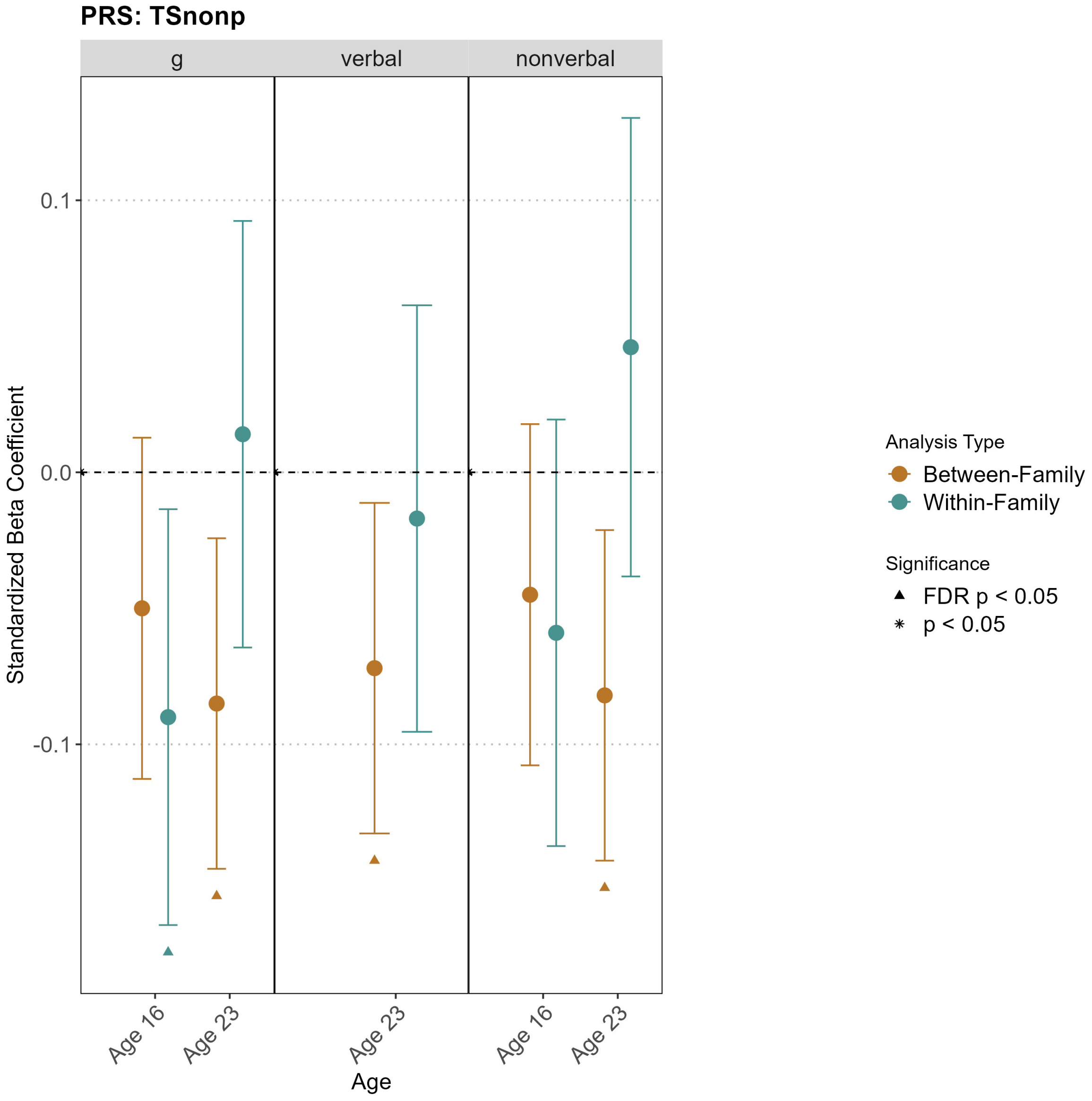

The figure presents the direct genetic effects and family-mediated indirect genetic effects of Tourette syndrome polygenic scores (corrected for p factor) on cognitive abilities across development.
